# Stable distinct core eukaryotic viromes in different mosquito species from Guadeloupe, using single mosquito viral metagenomics

**DOI:** 10.1186/s40168-019-0734-2

**Published:** 2019-08-28

**Authors:** Chenyan Shi, Leen Beller, Ward Deboutte, Kwe Claude Yinda, Leen Delang, Anubis Vega-Rúa, Anna-Bella Failloux, Jelle Matthijnssens

**Affiliations:** 1 KU Leuven Department of Microbiology, Immunology and Transplantation, Rega Institute, Laboratory of Viral Metagenomics, Leuven, Belgium; 2 KU Leuven Department of Microbiology, Immunology and Transplantation, Rega Institute, Laboratory of Virology and Chemotherapy, Leuven, Belgium; 3 Institut Pasteur of Guadeloupe, Laboratory of Vector Control Research, Unit Transmission, Reservoirs and Pathogen Diversity, Les Abymes, Guadeloupe; 4 Institut Pasteur, Department of Virology, Arboviruses and Insect Vectors, 25 rue du Dr Roux, 75724 Paris Cedex 15, France; 5 Laboratory of Virology, Rocky Mountain Laboratories, Division of Intramural Research, National Institute of Allergy and Infectious Diseases, National Institutes of Health, Hamilton, MT USA

**Keywords:** Viral metagenomics, Single mosquito, Eukaryotic virome, Phageome, Core virome, Guadeloupe, *Aedes aegypti*, *Culex quinquefasciatus*

## Abstract

**Background:**

Mosquitoes are the most important invertebrate viral vectors in humans and harbor a high diversity of understudied viruses, which has been shown in many mosquito virome studies in recent years. These studies generally performed metagenomics sequencing on pools of mosquitoes, without assessment of the viral diversity in individual mosquitoes. To address this issue, we applied our optimized viral metagenomics protocol (NetoVIR) to compare the virome of single and pooled *Aedes aegypti* and *Culex quinquefasciatus* mosquitoes collected from different locations in Guadeloupe, in 2016 and 2017.

**Results:**

The total read number and viral reads proportion of samples containing a single mosquito have no significant difference compared with those of pools containing five mosquitoes, which proved the feasibility of using single mosquito for viral metagenomics. A comparative analysis of the virome revealed a higher abundance and more diverse eukaryotic virome in *Aedes aegypti*, whereas *Culex quinquefasciatus* harbors a richer and more diverse phageome. The majority of the identified eukaryotic viruses were mosquito-species specific. We further characterized the genomes of 11 novel eukaryotic viruses. Furthermore, qRT-PCR analyses of the six most abundant eukaryotic viruses indicated that the majority of individual mosquitoes were infected by several of the selected viruses with viral genome copies per mosquito ranging from 267 to 1.01 × 10^8^ (median 7.5 × 10^6^) for *Ae*. *aegypti* and 192 to 8.69 × 10^6^ (median 4.87 × 10^4^) for *Cx*. *quinquefasciatus*. Additionally, in *Cx*. *quinquefasciatus*, a number of phage contigs co-occurred with several marker genes of *Wolbachia* sp. strain wPip.

**Conclusions:**

We firstly demonstrate the feasibility to use single mosquito for viral metagenomics, which can provide much more precise virome profiles of mosquito populations. Interspecific comparisons show striking differences in abundance and diversity between the viromes of *Ae*. *aegypti* and *Cx*. *quinquefasciatus*. Those two mosquito species seem to have their own relatively stable "core eukaryotic virome", which might have important implications for the competence to transmit important medically relevant arboviruses. The presence of *Wolbachia* in *Cx*. *quinquefasciatus* might explain (1) the lower overall viral load compared to *Ae*. *aegypti*, (2) the identification of multiple unknown phage contigs, and (3) the difference in competence for important human pathogens. How these viruses, phages, and bacteria influence the physiology and vector competence of mosquito hosts warrants further research.

**Electronic supplementary material:**

The online version of this article (10.1186/s40168-019-0734-2) contains supplementary material, which is available to authorized users.

## Background

Guadeloupe is the largest island of the French West Indies in the Caribbean, with an estimated population of 405,000 [1]. It features various landforms with a volcanic relief, rolling hills, and flat plains, attracting thousands of tourists annually from around the world [1]. However, the tropical climate and a half year rainy season facilitate efficient reproduction of mosquitoes and the viruses they carry. Viruses transmitted between animals and humans or among humans by insects or arachnids are referred to as arboviruses (arthropod-borne viruses), including the mosquito-borne viruses. During the last decades, the morbidity and mortality of mosquito-borne viruses placed a considerable burden on the healthcare system of Guadeloupe. Since the outbreak of dengue in 1994, this virus has been endemo-epidemic in Guadeloupe, with noticeable seasonal variation [2]. Co-circulation of several serotypes has also been observed. In 2010, Guadeloupe experienced a historical outbreak of dengue fever, which infected almost 10% of the population [2]. With shorter intervals and more sporadic cases between epidemic periods, as well as an increasing number of hospitalized cases, the epidemiology of dengue is evolving toward hyperendemicity [3]. Additionally, subsequent to the several imported cases of chikungunya in late 2005 and early 2006, an epidemic occurred in 2014 with more than 80,000 suspected clinical cases, followed by detection of autochthonous cases in 2016 and 2017 [4, 5]. Recently, Guadeloupe was also affected by the emergence of Zika. Approximately 31,000 cases have been reported up until June 2017, including 13 cases of Congenital Zika syndrome [6]. Co-infection of dengue-zika or dengue-chikungunya viruses might also occur in some regions. Furthermore, also yellow fever is a potential threat for the Caribbean, due to the ongoing circulation of yellow fever virus in the neighboring country Brazil [7] and the wide distribution of its vector *Aedes aegypti* in the region. A recent study has also shown that *Ae*. *aegypti* in Guadeloupe is susceptible to yellow fever virus [8]. Hence, with the population mobility among islands, population growth, and uncontrolled urbanization, the Caribbean region is under increasing risk of mosquito-borne viruses and hence forecasting the occurrence of epidemics is a challenge [2].

As it has been shown in several mosquito virome studies in recent years, mosquitoes harbor a high diversity of known and novel viruses [9–14]. Although most of these viruses are referred to as insect-specific viruses (ISVs), which have a restricted host range and do not replicate in vertebrate cells, they are highly prevalent in nature and usually belong to viral families also containing major mosquito-transmitted human pathogens, like *Flaviviridae*, *Bunyaviridae*, *Rhabdoviridae*, *Reoviridae*, or *Togaviridae* [15]. Increasing evidence suggest that ISVs might influence the mosquito physiology as well as its ability to transmit important arboviruses [16], which may provide a new avenue for biological vector control as well as novel vaccine platforms [17]. Although many bacteria have been reported to be involved in mosquito development and physiology as well [18, 19], their phages are studied far less, making this an interesting component of the mosquito virome for further studies.

Considering the tiny size and huge population of mosquitoes, previous studies generally performed metagenomics sequencing on pools of 15 to 50 mosquitoes [9–14]. However, this approach cannot show if a particular virome profile is representative for an individual mosquito, or if the virome profile is strongly skewed by one or a few acutely infected individuals with high viral titers. Additionally, both *Ae*. *aegypti* known as the key vector of chikungunya, dengue, and Zika viruses [20], and *Culex quinquefasciatus* which plays a significant role in West Nile virus transmission [21], are present across the entire Caribbean region. Both are urban mosquitoes colonizing domestic containers; *Ae*. *aegypti* mosquitoes breed mainly in clean water while *Cx*. *quinquefasciatus* prefer water with organic matter. A better understanding of the “commensal virome” in both mosquito species in Guadeloupe could lay the ground works for a better assessment of mosquito-borne disease risk, vector competence, and provide enlightenment on mosquito control.

Therefore, our study performed viral metagenomics sequencing on individual and pooled *Ae*. *aegypti* and *Cx*. *quinquefasciatus* collected from Guadeloupe in 2016 and 2017. Comparative analysis of eukaryotic virome and phageome were conducted between gender, location, and mosquito species. Several novel viruses were identified, and subsequently used for phylogenetic analyses and qRT-PCRs analyses to investigate possible core viruses in the mosquito population. Correlation analysis was used to identify the relationship between phage contigs and bacterial marker genes.

## Results

**Fig. 1:**
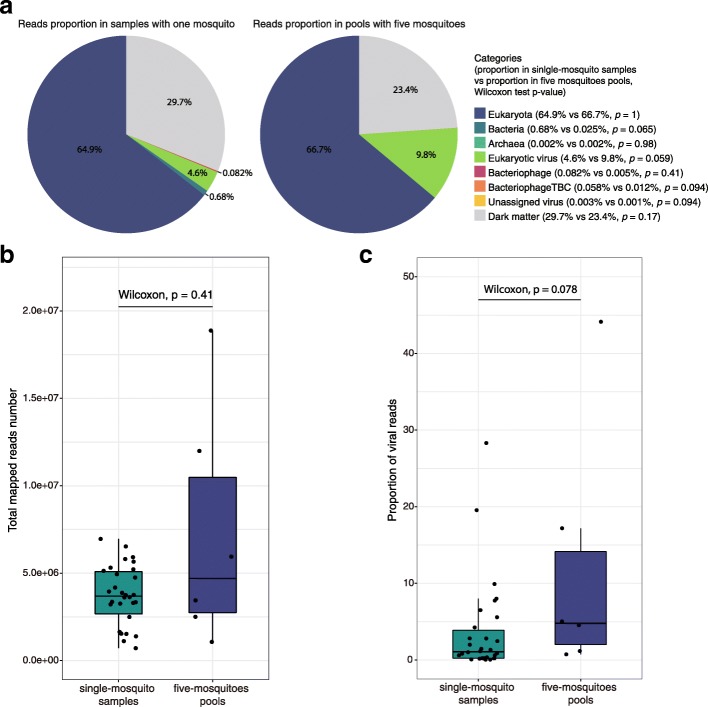
Comparison between NGS reads of single mosquito and pooled mosquitoes. **a** Proportion of each taxonomic category in single mosquito and pooled mosquitoes based on reads number. Legend contains the percentage of each category, as well as the *p* values of Wilcoxon test on the proportion of each category between single mosquito and pooled mosquitoes. **b** Comparison of total reads number mapped to the nr contigs collection in single mosquito and pooled mosquitoes. The nr contigs collection were obtained by removing the redundancy at 95% nucleotide identity over 80% of the length from all the de novo assembled contigs (> 500 bp) of all 36 samples. **c** Comparison of viral reads proportion (eukaryotic viruses, phages and unassigned virus) in single mosquito and pooled mosquitoes

Four pools containing males or females *Ae*. *aegypti* or *Cx*. *quinquefasciatus* collected from Les Abymes within the east island of Guadeloupe during the rainy season of 2016 were sequenced as a pilot study (Table 1, Additional file 1). The obtained (nearly) complete eukaryotic viral genomes were used for phylogenetic analyses (*vide infra*). Furthermore, additional samples were collected from Les Abymes and Petit-Bourg (in the west island of Guadeloupe) in 2017 (Additional file 1). For each mosquito species, gender and sampling location, five individual mosquitoes, and one pool with five mosquitoes (total 36 samples) were prepared and sequenced (Table 1). For the 36 samples, an average of 7 million NGS reads per sample were obtained after trimming and decontamination (Additional file 2), and subsequently de novo assembled into 2,657,612 contigs. After the removal of all contigs shorter than 500 bp (94.5%), the remaining contigs were filtered for redundancy at 95% nucleotide identity over 80% of the length, resulted in 75,213 non-redundant (nr) contigs from all samples. This nr contigs set was taxonomically annotated using BLASTn, DIAMOND, as well as VirSorter and MetaPhinder2 to identify highly divergent phages. Finally, they were separated into eight categories: Eukaryota, Bacteria, Archaea, eukaryotic virus, bacteriophage, bacteriophage to be confirmed (bacteriophageTBC, *vide infra*), unassigned virus, and dark matter (Fig. 1a). Ninety-two and twelve contigs were annotated as eukaryotic virus and unassigned virus, respectively, whereas 299 contigs were predicted to be of bacteriophage origin. Hmmsearch against the Prokaryotic Virus Orthologous Groups (pVOGs), eggNOG-mapper, and PfamScan were further used to confirm the bacteriophage contigs. Out of these 299 contigs, 105 contigs showed neither pVOGs hits nor phage-associated protein/domain/motif, and were therefore classified as bacteriophageTBC. The dark matter included the contigs that got no significant hits from DIAMOND (BLASTx), BLASTn, or phage identification software (VirSorter and MetaPhinder2).

**Table 1:** Pooling information of mosquitoes before sequencing

Year	Location	Mosquito species	Gender	Pools	Abbreviation
2016	Les Abymes	*Aedes aegypti*	Female	1 pool with 24 mosquitoes	Ab-AAF
			Male	1 pool with 21 mosquitoes	Ab-AAM
		*Culex quinquefasciatus*	Female	1 pool with 30 mosquitoes	Ab-CQF
			Male	1 pool with 20 mosquitoes	Ab-CQM
2017	Les Abymes	*Aedes aegypti*	Female	5 pools with 1 mosquito	Ab-AAF-1-1~5
				1 pool with 5 mosquitoes	Ab-AAF-5
			Male	5 pools with 1 mosquito	Ab-AAM-1-1~5
				1 pool with 5 mosquitoes	Ab-AAM-5
	Petit-Bourg	*Aedes aegypti*	Female	5 pools with 1 mosquito	PB-AAF-1-1~5
				1 pool with 5 mosquitoes	PB-AAF-5
			Male	5 pools with 1 mosquito	PB-AAM-1-1~5
				1 pool with 5 mosquitoes	PB-AAM-5
		*Culex quinquefasciatus*	Female	5 pools with 1 mosquito	PB-CQF-1-1~5
				1 pool with 5 mosquitoes	PB-CQF-5
			Male	5 pools with 1 mosquito	PB-CQM-1-1~5
				1 pool with 5 mosquitoes	PB-CQM-5

## Feasibility of viral metagenomic on individual mosquitoes

Figure 1a shows the proportion of each taxonomic category in single-mosquito samples and five-mosquitoes pools based on the reads number (Additional file 2). The majority of the reads in both the single-mosquito samples (64.9%) and five-mosquitoes pools (66.7%) were found to be Eukaryota and were mainly derived from the mosquito host genome (Fig. 1a). The percentage of eukaryotic virus reads in the single-mosquito samples was lower than that in the five-mosquitoes pools, whereas the bacteria, bacteriophage, and bacteriophageTBC proportion in the single-mosquito pools was higher compared to the five-mosquitoes pools (Fig. 1a). However, none of these differences was significant between any category of single-mosquito samples and five-mosquitoes pools (Fig. 1a).

In the 30 single-mosquito samples, 708,000 to 6 million reads per sample were aligned to the nr contigs set with a median of 3.69 million reads. One million to 18 million reads per five-mosquitoes pool were aligned to the nr contigs set with a median of 4.7 million reads (Fig. 1b). The aligned reads number between both groups was not statistically significant (Wilcoxon test, *p* value = 0.41). The proportion of the viral reads (reads mapped to eukaryotic virus, bacteriophage, and unassigned virus contigs) per sample in single-mosquito samples vs. five-mosquitoes pools was also not significantly different (Wilcoxon test, *p* value = 0.078), although a median proportion of 1% in the single-mosquito pools and 4.8% in the five-mosquitoes pools were found (Fig. 1c).

## Overview of eukaryotic virome and phageome in two mosquito species

**Fig. 2:**
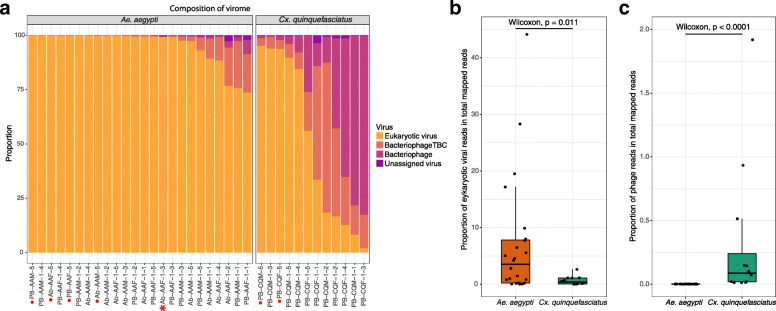
Comparison between viral reads in *Aedes aegypti* and *Culex quinquefasciatus* per sample/pool. **a** Proportion of eukaryotic virus, bacteriophage, bacteriophageTBC, and unassigned virus in each sample/pool, for *Aedes aegypti* and *Culex quinquefasciatus*. The samples are ranked in a descending proportion of eukaryotic virus reads. The samples marked with red dots are pools containing five mosquitoes, whereas the other samples contain individual mosquitoes. Samples Ab-AAF-1-3 is labeled with a star symbol. **b** Comparison of the proportion of eukaryotic virus reads in the two mosquito species. **c** Comparison of the proportion of bacteriophage reads in the two mosquito species

Eukaryotic viruses occupied the vast majority of viral reads in *Ae*. *aegypti* samples/pools, whereas half of the *Cx*. *quinquefasciatus* samples/pools were dominated by bacteriophages (Fig. 2a). Further comparative analysis between these two species revealed that *Ae*. *aegypti* samples possessed a significantly higher percentage of eukaryotic virus reads compared to *Cx*. *quinquefasciatus* (Wilcoxon test, *p* value = 0.011, Fig. 2b), whereas the opposite was observed for the bacteriophages (Wilcoxon test, *p* value = 1.5e-06, Fig. 2c). For the other taxonomic categories, the proportion of bacteria, bacteriophageTBC, and unassigned virus were also significantly higher in *Cx*. *quinquefasciatus* with *p* value < 0.0001 of Wilcoxon test (Additional file 3C, 3D, 3E).

**Fig. 3:**
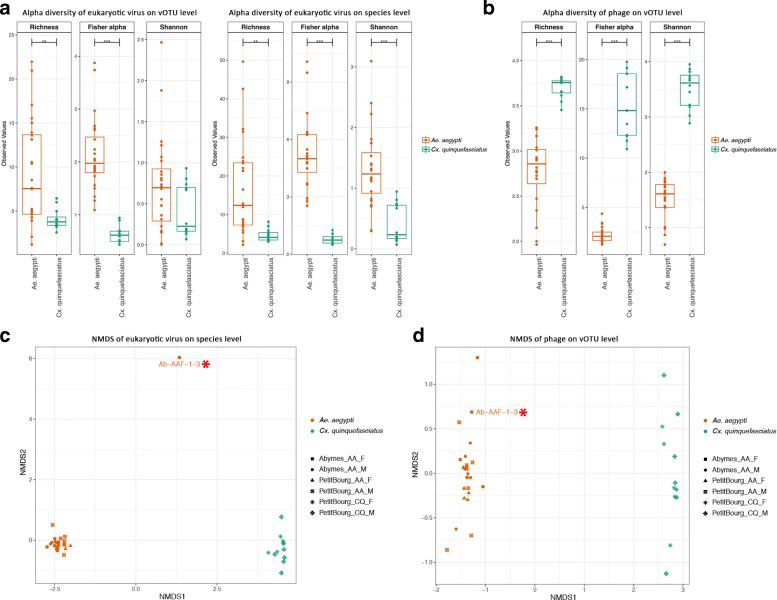
Alpha and beta diversity of the virome in *Aedes aegypti* and *Culex quinquefasciatus* samples/pools. **a** Alpha diversity of eukaryotic viruses in *Aedes aegypti* and *Culex quinquefasciatus* on vOTU and species level. **b** Alpha diversity of bacteriophage contigs in *Aedes aegypti* and *Culex quinquefasciatus* on vOUT level. Pairwise ANOVA: *p* < 0.01 (*), *p* < 0.001 (**), *p* < 0.0001 (***). **c** Non-metric multi-dimensional scaling (NMDS) of eukaryotic viruses on viral species level. Samples Ab-AAF-1-3 is labeled with text and a star symbol. STRESS = 0.0425, PERMANOVA test on mosquito species: *p* = 0.001, R^2^ = 0.126. **d** NMDS of bacteriophages on vOTU level. Samples Ab-AAF-1-3 is labeled with text and a star symbol. STRESS = 0.034, PERMANOVA test on mosquito species: *p* = 0.001, R^2^ = 0.311

These observations were further confirmed by significant higher richness, Fisher and Shannon indices of the eukaryotic virome in *Ae*. *aegypti* compared to *Cx*. *quinquefasciatus* on viral species and viral OTU (vOTU) levels (except for the Shannon value on the vOTU level) (Fig. 3a). There was no significant difference found between gender or locations within the *Ae*. *aegypti* population (Additional file 4). The richness and Fisher indices were significantly higher in the *Cx*. *quinquefasciatus* females than the males (Additional file 4B). Because most phage contigs were identified using VirSorter or MetaPhinder2, without nucleotide or amino acid similarity to known taxonomically classified phages, the alpha diversity analysis of the phageome was only done on the vOTU level. In sharp contrast to the eukaryotic virome, alpha diversity indices of the phageome in *Cx*. *quinquefasciatus* were remarkably higher than for *Ae*. *aegypti* (Fig. 3b). For the beta diversity, Bray-Curtis dissimilarities were calculated from the abundance of eukaryotic viral species or bacteriophage vOTUs and then used for unconstrained ordination analysis with non-metric multi-dimensional scaling (NMDS). A clear separation of eukaryotic viral and phage communities according to the mosquito species was evident in Fig. 3c, d, respectively. Permutational multivariate analysis of variance (PERMANOVA) test on mosquito species resulted in *p* = 0.001 and R^2^ = 0.126 for the eukaryotic virome and *p* = 0.001 and R^2^ = 0.311 for the phageome, further suggesting that the viromes in these two mosquito species had different centroids. Notably, the eukaryotic virome of a specific sample Ab-AAF-1-3 (a female adult *Ae*. *aegypti* collected in Les Abymes) neither clustered with *Ae*. *aegypti* nor *Cx*. *quinquefasciatus*, whereas its phageome clustered within the *Ae*. *aegypti* population, which only contained very few (20 out of 194 contigs identified) confirmed phage contigs. This result together with other data (*vide infra*) suggests that the specific sample Ab-AAF-1-3 belonged to another mosquito species rather than *Ae*. *aegypti*. So, the virome comparison analysis shown in Figs. 2b, c and 3 were repeated after removing the sample Ab-AAF-1-3 from the *Ae*. *aegypti* group (Additional file 3A and 3B, Additional file 5), resulting in very similar results and the same conclusions.

## Eukaryotic virome

**Fig. 4:**
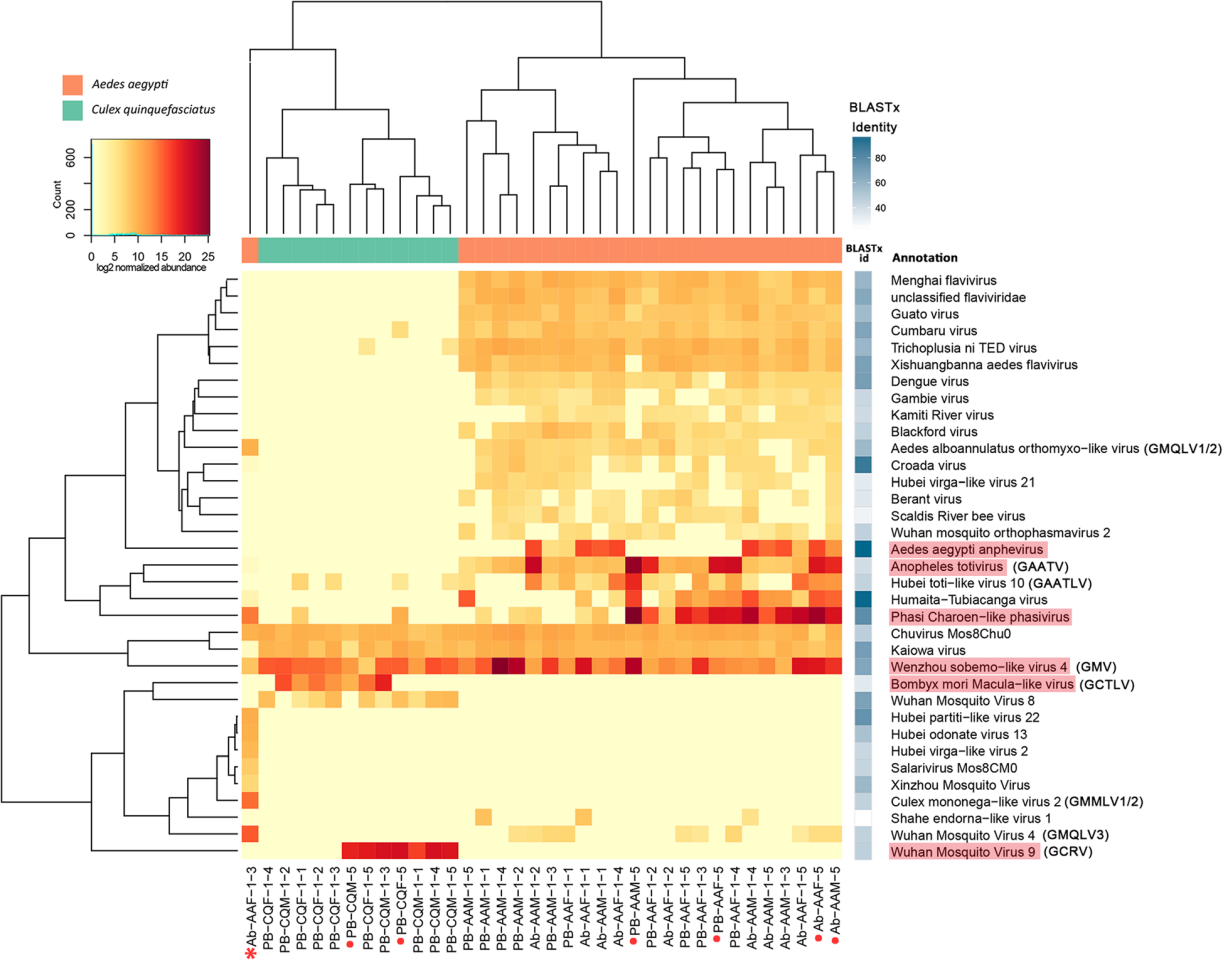
Normalized abundance of eukaryotic viral species. The heatmap shows the normalized reads counts by metagenomeSeq on log2 scale. The hierarchical clustering is based on the Euclidean distance matrix calculated from the normalized reads count. The viral species names shown in the heatmap are from the taxonomic annotation by DIAMOND and KronaTools. For each of the contigs assigned to a particular species, the ORF with the highest BLASTx identity to a reference sequence was taken, and the average identity of these different ORFs is shown in the shaded blue boxes. The red-shaded viruses were selected for qRT-PCR analysis and the names of novel viruses are shown between brackets. The samples marked with red dots are pools containing five mosquitoes and the one with star is the special sample Ab-AAF-1-3

The different pattern of the eukaryotic virome between *Ae*. *aegypti* and *Cx*. *quinquefasciatus* was also evident in log2 normalized abundance of 35 eukaryotic viral species (rows) across the 36 samples/pools as shown in Fig. 4. Two viral species that had less than 50 reads were removed from the analysis. The virus names shown in the heatmap were from the taxonomic annotation of DIAMOND and KronaTools based on BLASTx. Sometimes the viruses identified in our study were quite divergent from these viral species, as shown by the different shades of blue squares. The viromes of *Ae*. *aegypti* and *Cx*. *quinquefasciatus* samples/pools clearly clustered separately according to the hierarchical clustering based on the Euclidean distance matrix, except for the previously mentioned sample Ab-AAF-1-3, which formed a separate clade, characterized by a set of unique viruses. *Ae*. *aegypti* and *Cx*. *quinquefasciatus* had a few viruses in common, such as Wenzhou sobemo-like virus 4 with a high abundance, and Chuvirus Mos8Chu0 and Kaiowa virus with a lower abundance. Reads of Phasi Charoen-like phasivirus and Hubei toti-like virus 10 were highly abundant in *Ae*. *aegypti*, and only sporadically presented in *Cx*. *quinquefasciatus*, suggesting a lower viral load in *Cx*. *quinquefasciatus*. Some viruses were uniquely present in *Ae*. *aegypti* (e.g., Aedes aegypti anphevirus and Anopheles totivirus) or *Cx*. *quinquefasciatus* (e.g., *Bombyx mori* Macula-like virus and Wuhan Mosquito Virus 9). Several short contigs (less than 1000 bp) were assigned to mosquito-specific flaviviruses, like Menghai flavivirus and Xishuangbanna aedes flavivirus. Interestingly, one 757 bp contig was found to have 71% aa identity with the NS5 region of Dengue virus 2 and 69% with that of Kamiti river virus. Considering the high conservation of the NS5 gene in the family *Flaviviridae*, the contig annotated as Dengue virus might be the partial genome of a novel mosquito-specific flavivirus or an endogenous viral element deriving from non-retroviral RNA virus (e.g., Kamiti river virus) [22, 23]. In addition to a few of the viral species highly abundant in *Ae*. *aegypti*, the distinctive sample Ab-AAF-1-3 also possessed a group of unique viruses, such as Culex Mononega-like virus 2.

## Further characterization of novel viruses

Several viruses for which a near complete genome (at least the complete coding regions) could be identified were selected for further phylogenetic analysis. The names and abbreviations of the novel viruses and their taxonomic annotation by DIAMOND and KronaTools are shown in Table 2. Furthermore, the obtained viral genome length and accession number of each species identified in this study as well as the name, genome length, and accession number of their most closely related reference genomes are shown in Additional file 6. Interestingly, several of these viruses were identified in both 2016 and 2017, as well as in both locations (Fig. 4, Additional file 7).

**Table 2:** Novel viruses identified in this study

Virus taxon	Novel viruses	Abbreviation	Taxonomic annotation by DIAMOND and KronaTools
*Luteoviridae & Sobemovirus*	Guadeloupe mosquito virus	GMV	Wenzhou sobemo-like virus 4
			Hubei mosquito virus 2
*Phasivirus*	Guadeloupe mosquito phasivirus	GMPV	Phasi Charoen-like phasivirus
*Totiviridae*	Guadeloupe Aedes aegypti totivirus	GAATV	Anopheles totivirus
	Guadeloupe Aedes aegypti toti-like virus	GAATLV	Hubei toti-like virus 10
Mononegavirales	Guadeloupe mosquito mononega-like virus 1	GMMLV1	Culex mononega-like virus 2
	Guadeloupe mosquito mononega-like virus 2	GMMLV2	Culex mononega-like virus 2
*Quaranjavirus*	Guadeloupe mosquito quaranja-like virus 1	GMQLV1	Aedes alboannulatus orthomyxi-like virus
	Guadeloupe mosquito quaranja-like virus 2	GMQLV2	Aedes alboannulatus orthomyxi-like virus
	Guadeloupe mosquito quaranja-like virus 3	GMQLV3	Wuhan Mosquito Virus 4
*Rhabdoviridae*	Guadeloupe Culex rhabdovirus	GCRV	Wuhan Mosquito Virus 9
*Tymoviridae*	Guadeloupe Culex tymo-like virus	GCTLV	*Bombyx mori* Macula-like virus

### *Luteoviridae* and *Sobemovirus*-related viruses

**Fig. 5:**
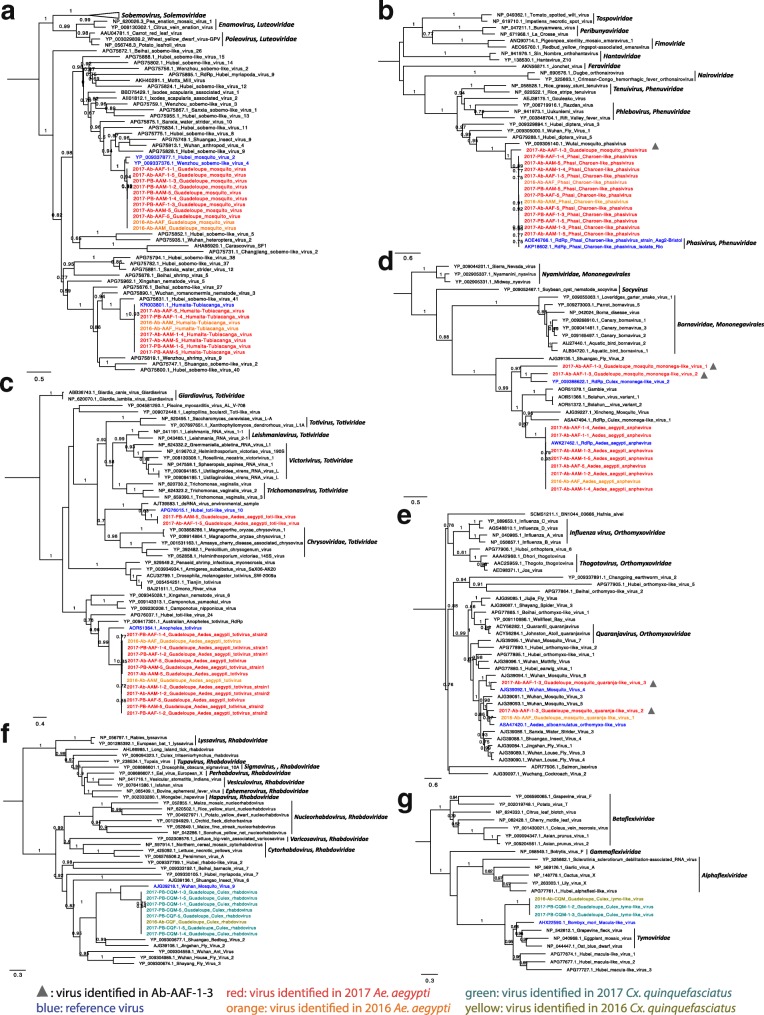
Phylogenetic trees of selected eukaryotic viruses identified in 2016 and 2017 samples. **a** ML phylogeny of *Luteoviridae* and *Sobemovirus*-related viruses based on amino acid sequence of RdRp. **b** ML phylogeny of *Phasivirus-*related viruses based on amino acid sequence of RdRp. **c** ML phylogeny of *Totiviridae-*related viruses based on amino acid sequence of RdRp. **d** ML phylogeny of Mononegavirales-related viruses based on amino acid sequence of RdRp. **e** ML phylogeny of *Quaranjavirus-*related viruses based on the amino acid sequence of PB1. **f** ML phylogeny of *Rhabdoviridae-*related viruses based on amino acid sequence of RdRp. **g** ML phylogeny of *Tymoviridae-*related viruses based on amino acid sequence of RdRp. The most closely related references are in blue. Viruses identified from *Aedes aegypti* in 2016 and 2017 are orange and red, respectively. Viruses identified from the unique sample Ab-AAF-1-3 are marked with a gray triangle. Viruses identified from *Culex quinquefasciatus* in 2016 and 2017 are in light green and dark green, respectively

In recent years, a wide range of highly divergent viruses have been identified distantly related from the ICTV family *Luteoviridae* and genus *Sobemovirus*. Although viruses belonging to this family/genus were believed to be plant viruses with a monopartite genome, many of these novel viruses had (bi) segmented genomes [24]. The closest relatives of Guadeloupe mosquito virus (GMV) identified in our study were Wenzhou sobemo-like virus 4 (WSLV4) and Hubei mosquito virus 2 (HMV2). The RNA-dependent RNA polymerase (RdRp) segment of GMV was closely related to WSLV4 (86% similarity on the amino acid level) with a similar genome organization (Additional file 8A). The capsid-encoding segment of WSLV4 is missing, and therefore segment 2 of GMV was most closely related to the HMV2 (49% amino acid identity) reference strain. In the RdRp phylogeny, GMVs from pools of 2016 and 2017 formed a new clade that differed from WSLV4 and HMV2 (Fig. 5a). The two segments of Humaita-Tubiacanga virus (HTV) identified in 2016 and 2017 were very closely related to the reference (99% amino acid identity, Fig. 5a), which has only been described in *Ae*. *aegypti* from Brazil [25]. No variations were observed between viruses identified in 2016 and 2017.

### *Phasivirus*-related viruses

Phasi Charoen-like phasivirus (PCLPV) belongs to the recently created genus *Phasivirus* in the new family *Phenuiviridae* of the new order *Bunyavirales* (https://talk.ictvonline.org/files/ictv_official_taxonomy_updates_since_the_8th_report/). Its genome contains three segments (S, M, and L) as most other bunyaviruses. Due to the low abundance of PCLPV in *Cx*. *quinquefasciatus*, no complete segments were obtained. However, all three segments of PCLPV genome were found in 50% of *Ae*. *aegypti* samples/pools sequenced in 2017 (Fig. 5b). Most of PCLPVs identified in 2016 and 2017 samples had very close relationship with the references (99% amino acid identity of RdRp). The unusual sample Ab-AAF-1-3 contained a distantly related virus named as Guadeloupe mosquito phasivirus (GMPV) (Table 2, Fig. 5b), only showing 66% amino acid identity of RdRp (L), 55% of glycoprotein (M), and 58% of capsid (S) with PCLPV.

### *Totiviridae*-related viruses

The RdRp gene of Guadeloupe Aedes aegypti totivirus (GAATV) was slightly shorter than its closest relative Anopheles totivirus (Additional file 8B) and showed 45% amino acid identity to that of Anopheles totivirus. In addition, a 471 aa open reading frame (ORF) before the capsid coding region without known function was found unexpectedly in almost half of assembled GAATV genomes. Interestingly, sometimes more than one GAATV genome was identified within a single-mosquito sample or a five-mosquitoes pool. In the phylogenetic tree based on the RdRp of GAATVs and other *Totiviridae-*related viruses, two slightly divergent variants of GAATVs were observed, which formed two separate clusters (Fig. 5c, Additional file 9). Further analysis, on the RdRp and Capsid proteins of GAATVs, showed different topological structure (Additional file 9). For instance, the RdRp of GAATVs identified in 2016 fell into two clusters, whereas their capsid proteins fall into a single cluster. This indicated possible recombination events among these viruses. Additionally, Guadeloupe Aedes aegypti toti-like virus (GAATLV) identified in *Ae*. *aegypti* in 2017 was divergent from Hubei toti-like virus 10 with only 52% amino acid identity of RdRp (Fig. 5c).

### Mononegavirales-related viruses

Aedes aegypti anpheviruses (AANV) identified in both *Ae*. *aegypti* samples of 2016 and 2017 had 99% aa identity with the reference in GenBank. In addition, we identified two distantly related Guadeloupe mosquito mononega-like viruses (GMMLV) in the unusual mosquito (Ab-AAF-1-3). These GMMLV1 and GMMLV2 sequences had 37% and 52% amino acid similarity with Culex mononega-like virus 2 (CMLV2), respectively. In the phylogenetic tree, GMMLV1 is located in a new clade, which was more distant from GMMLV2 and CMLV2 (Fig. 5d).

### *Quaranjavirus*-related viruses

The novel Guadeloupe mosquito quaranja-like virus 1, 2, and 3 (GMQLV1–3) belong to two separate clusters, which were related to the genus *Quaranjavirus* in the family *Orthomyxoviridae* (Fig. 5e). The genome of members in genus *Quaranjavirus* normally contains six to seven segments [26]. Only two segments (polymerase subunit PB1 and PB2) of GMQLV1 could be identified from a female *Ae*. *aegypti* pool of 2016. Although GMQLV2 and GMQLV3 reads were present in many *Ae*. *aegypti* pools, they were only highly abundant in the unusual sample Ab-AAF-1-3. Three near complete segments (PB1, PB2, and PA) of GMQLV2 and five segments (PB1, PB2, PA, NP, and GP) of GMQLV3 were identified in sample Ab-AAF-1-3. The PB1 sequences of GMQLV1 and GMQLV2 were related to Aedes alboannulatus orthomyxi-like virus (AAOLV, 66% and 67% amino acid similarity, respectively), which was recently found in *Ae*. *alboannulatus* from West Australia [27]. GMQLV3 clusters together with Wuhan Mosquito Virus 4 (WMV4, 67% amino acid similarity of PB1) and Wuhan Mosquito Virus 6 (WMV6, 54% amino acid similarity of PB1), both initially identified from *Culex* mosquitoes in China [24].

### *Rhabdoviridae*-related viruses

The novel virus Guadeloupe Culex rhabdovirus (GCRV) was specifically found in *Cx*. *quinquefasciatus* and phylogenetically distantly (46% amino acid identity of RdRp) related to Wuhan mosquito virus 9 (WMV9) within the family *Rhabdoviridae* (Fig. 5f). The RdRp of WMV9 consisted of two separate ORFs, whereas our GCRV had a longer and presumably complete RdRp ORF (Additional file 8C).

### *Tymoviridae*-related viruses

The genome size of the novel Guadeloupe Culex tymo-like virus (GCTLV) was approximately 2000 bp longer than its closest relative *Bombyx mori* Macula-like virus (BmMLV) isolated from the BmN cell line [28]. Besides the capsid and longer RdRp genes, the GCTLV genome also contained a small additional ORF at its 3′ end without known function (Additional file 8D). The three identified GCTLV strains clustered together in a distinct clade, separated from other reference strains (Fig. 5g). Although the family *Tymoviridae* are plant viruses, many of the virus strains related to this family have been discovered from spider, Odonata, or insect cell, suggesting that the *Culex* mosquito might be the true host of GCTLV.

## qRT-PCR confirmation of core virome

**Fig. 6:**
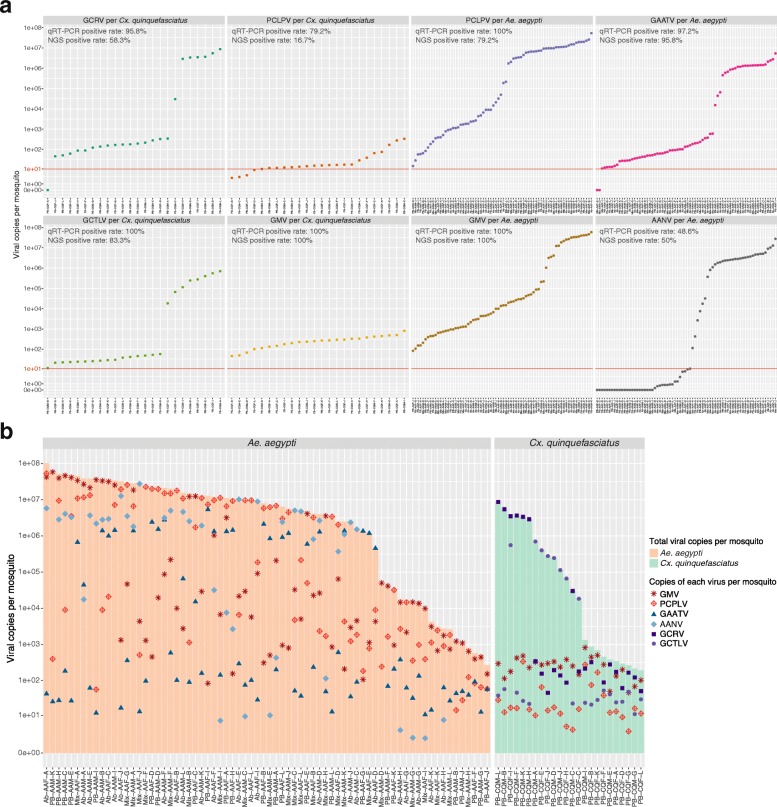
Quantification of GMV, PCPLV, AANV, GAATV, GCLTV, and GCRV in mosquito populations. **a** Copy number of each screened virus in individual *Aedes aegypti* or *Culex quinquefasciatus*. *Y*-axis is in log scale. The red lines indicate the ten copies, which was used as threshold to calculate the positive rate. The NGS positive rates are calculated from the reads abundance, using one read as threshold. **b** Total viral genome copies in each individual mosquito. The light orange and green bars indicate the total viral genome copies per individual of *Aedes aegypti* and *Culex quinquefasciatus*, respectively. Six different symbols with difference colors indicate the genome copies of each detected viruses

No major quantitative claims can be made from viral metagenomics shotgun data, due to its relative nature. Therefore, we designed quantitative real-time RT-PCR (qRT-PCR) primers, probes, and quantification standards to quantify a selection of six viruses (Additional file 10). We selected the two most abundant viruses present in both mosquito species (PCLPV and GMV), as well as two *Ae*. *aegypti*-specific (GAATV and AANV) and two *Cx*. *quinquefasciatus*-specific (GCRV and GCTLV) eukaryotic viral species. Thus, four viruses were measured for each mosquito species in additional individual mosquito samples from the 2017 collection expedition (Table 3). In addition to the samples from Les Abymes and Petit-Bourg, a group of *Ae*. *aegypti* mosquitoes collected at multiple locations of Guadeloupe were also included in the qRT-PCR screening. In total, the copy numbers of these viruses were determined in 72 *Ae*. *aegypti* and 24 *Cx*. *quinquefasciatus* individuals. Ten copies of each virus per mosquito sample were used as an arbitrary threshold to calculate the positivity rate. It was impressive to detect GMV in all 96 tested samples (of both species), and PCPLV in all tested *Ae*. *aegypti* samples and 79.2% of *Cx*. *quinquefasciatus* samples (Fig. 6a). However, dramatically higher number of genome copies of PCLPV and GMV were found in *Ae*. *aegypti* (5.32 × 10^7^ and 5.85 × 10^7^ as maximum copy numbers, respectively) compared to *Cx*. *quinquefasciatus* individuals (with 336 and 816 copies maximally, respectively). For *Cx*. *quinquefasciatus*-specific viruses, 95.8% and 100% of *Culex* individuals were positive for GCRV and GCTLV, respectively (Fig. 6a). The maximum concentration of these viruses was 8.69 × 10^6^ and 7.02 × 10^5^ copies per individual, respectively. GAATV and AANV were found present in 97.2% and 48.6% of *Ae*. *aegypti* samples, which was comparable to the NGS results (23/24 and 12/24, Figs. 6a and 4). The detected highest viral load of these viruses reached up to 5.36 × 10^6^ and 2.75 × 10^7^, respectively. In general, the total number of genome copies of the selected viruses per mosquito ranged from 267 to 1.01 × 10^8^ (with a median of 7.5 × 10^6^) in *Ae*. *aegypti*, and from 192 to 8.69 × 10^6^ (with a median of 4.87 × 10^4^) in *Cx*. *quinquefasciatus* individuals (Fig. 6b). The observed lower viral load in *Cx*. *quinquefasciatus* compared to *Ae*. *aegypti* confirmed the observed NGS data (Fig. 2b), suggesting that the lower proportion of the eukaryotic virome in *Cx*. *quinquefasciatus* was not the result of the higher abundance of phages. Additionally, qRT-PCR results showed that some individuals (e.g., Ab-AAM-F and Mix-AAM-A) contained a high viral load for all four tested viruses, whereas other individuals (e.g., PB-AAF-J and PB-CQF-L) contained very low levels of all tested viruses (Fig. 6b). It should be noted that the detection of ten genome copies is rather arbitrarily and that the presence of nucleic acids does not prove replication. Especially low amounts of viral copies could potentially be remnants of a blood meal or vertical transmission.

**Table 3:** Individual mosquito samples (2017) used for qRT-PCR detection

Year	Location	Mosquito species	Gender	Detected mosquito no.	Abbreviation
2017	Les Abymes	*Aedes aegypti*	Female	12	Ab-AAF-A~L
			Male	12	Ab-AAM-A~L
	Petit-Bourg	*Aedes aegypti*	Female	12	PB-AAF-A~L
			Male	12	PB-AAM-A~L
		*Culex quinquefasciatus*	Female	12	PB-CQF-A~L
			Male	12	PB-CQM-A~L
	Multiple locations^a^	*Aedes aegypti*	Female	12	Mix-AAF-A~L
			Male	12	Mix-AAM-A~L

^a^Mosquito samples were collected around the downtowns of Petit-Bourg, Lamentin, Baie-mahault, Les Abymes, Saint François, and Saint Claude in Guadeloupe

## Marker genes identification

**Fig. 7:**
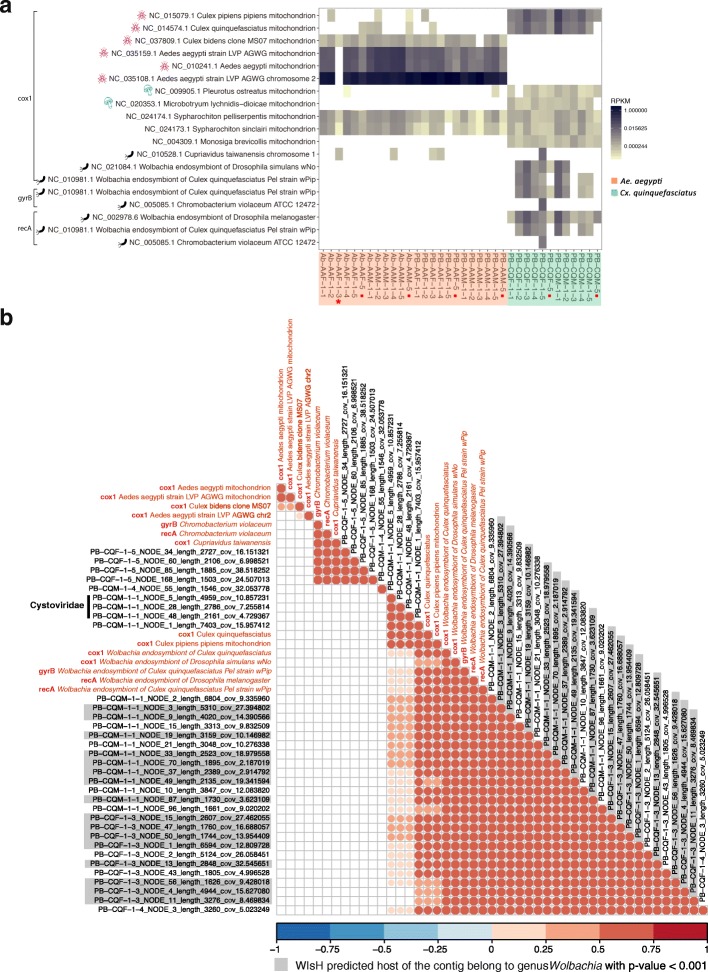
Marker genes identification and correlation analysis. **a** Heatmap of detected marker genes (cox1, gyrB, and recA) in NGS data of *Aedes aegypti* and *Culex quinquefasciatus* pools. The color of the heat map indicates the RPKM of the genes. The *Aedes aegypti* and *Culex quinquefasciatus* pools are highlighted with orange and green background, respectively. Pools containing five mosquitoes are marked with red dots and the sample marked with a star is the special sample Ab-AAF-1-3. **b** Correlation analysis on relative abundance of confirmed phage contigs (> 1500 bp), bacteria, and mosquito marker genes. The gradation of red color in the circle indicates the degree of positive correlation. The bigger size of the circle associates with lower *p* value. Only the correlations with an adjusted *p* value less than 0.01 are shown in the figure. The marker genes are labeled with red font color and phage contigs are labeled with black font color. Phage contigs of which WIsH predicted the genus *Wolbachia* as the host (*p* < 0.001) are marked in gray

Although our NetoVIR protocol was designed to purify virus particles from biological samples, it cannot avoid that genomic DNA of the host or bacteria survived our procedures (centrifugation/filtration/nuclease treatment) and was sequenced. These host-derived genomic reads (Additional file 2) allowed us to use molecular method as a confirmation of the mosquito species, which was especially useful for the sample Ab-AAF-1-3, possessing the distinct eukaryotic virome (Figs. 3c and 4). The trimmed and decontaminated reads of individual samples were mapped to the collection of all cytochrome c oxidase subunit 1 (cox1) genes (except cox1 genes of mammals) as a marker gene of eukaryota [29] and some prokaryota. Meanwhile, the DNA gyrase subunit B (gyrB) and recombinase A protein (recA) genes were used to identify the bacteria [30] in the samples. The marker genes whose sum reads per kilobase million (RPKM) value of all samples was higher than 0.001 were used for further analysis. All *Cx*. *quinquefasciatus* samples and pools contained a number of reads (ranging from six to 915) mapping against the *Cx*. *quinquefasciatus* and *Cx*. *pipiens* cox1 genes as could be expected (Fig. 7a). All the *Ae*. *aegypti* individual samples and pools except Ab-AAF-1-3 contained a large number of reads (ranging from 7699 to 294,803) mapping to the three *Ae*. *aegypti* cox1 genes (Fig. 7a). Except for samples Ab-AAF-1-3 and PB-CQF-5, all samples and pools had a high length coverage (70% to 100%) of the *Ae*. *aegypti* cox1 gene (NC_035159.1, 1537 bp) or *Cx. quinquefasciatus* cox1 gene (NC_014574.1, 1537 bp). Although 2,409,183 reads in the unusual sample Ab-AAF-1-3 were assigned to the mosquito genome, it had no reads mapping against the *Ae*. *aegypti* cox1 genes, and only a low background level of reads mapping against the *Cx. bidens* cox1 genes (as did all the true *Ae*. *aegypti* samples), suggesting that this mosquito belonged to a mosquito species whose cox1 gene was not present in databases. In addition, the cox1 genes of two fungi (*Microbotryum lychnidis-dioicae* and *Pleurotus ostreatus*) were also detected at low levels in *Cx*. *quinquefasciatus*. The cox1, gyrB, and recA genes of endosymbiotic bacteria *Wolbachia* sp. strain wPip were all found to be prevalent in *Cx*. *quinquefasciatus* samples and pools. Specifically, the sample PB-CQF-1-5 also possessed the marker genes of *Chromobacterium violaceum* and *Cupriavidus taiwanensis*, which are abundant components of the soil and water in tropical and subtropical regions [31, 32], and were therefore bacteria likely obtained from the environment.

## Correlation of bacteriophage vOTUs and bacteria genes

As mentioned before, the majority of the phage genomes were identified using VirSorter or MetaPhinder2 and had no recognizable nucleotide or amino acid similarity to known taxonomically classified phages, which did not allow us to speculate about their bacterial hosts. Therefore, we subjected the relative abundance of mosquito host marker genes, bacterial marker genes, and confirmed bacteriophage contigs longer than 1500 bp (33 contigs) to a correlation coefficient calculation (Fig. 7b). Multiple marker genes of *Ae*. *aegypti* and *Cx*. *bidens* clustered together, and none of the bacteriophage contigs correlated with them. Four bacteriophage contigs had highly and statistically significant correlation with marker genes of *Chromobacterium violaceum* and *Cupriavidus taiwanensis*. Twenty-four bacteriophage contigs were significantly correlated with the marker genes of *Wolbachia* sp. strain wPip and *Cx*. *quinquefasciatus*. Additionally, the three contigs classified as the L, M, and S segments of a member of the *Cystoviridae*, an additional 7403 bp contig as well as cox1 genes of *Cx*. *quinquefasciatus* clustered together. This suggested that the potential bacterial host of this phage was symbiotic in *Cx*. *quinquefasciatus*. Unfortunately, no bacterial marker genes could be detected, which might be due to the fact that no genomic DNA of this bacterium survived our procedures, or its marker genes are not present in databases. One of the natural hosts of *Cystoviridae* are members of the *Pseudomonas* genus (https://viralzone.expasy.org/165), commonly found in gut microbes of malaria mosquitoes [33], which suggests the potential existence of *Pseudomonas* bacteria in *Cx*. *quinquefasciatus*.

To further substantiate the prokaryotic host prediction of these phage genomic contigs, we used WIsH [34], a program that predicts the prokaryotic host of genomic phage contigs based on trained Markov models and k-mer frequencies. From their benchmark results, WIsH predicts hosts for 90% of the phage sequences (> 3kbp) with 80% accuracy at the genus level at a *p* value threshold of 0.001 [34]. Among the 33 phage contigs (from Fig. 7b), 16 contigs had a *p* value lower than 0.001 and all their predicted hosts belonged to the genus *Wolbachia* (Additional file 11), consistent with the correlation analysis (Fig. 7b). The WIsH-predicted host of the other eight contigs (which were correlated with *Wolbachia* marker genes in Fig. 7b), were also predicted to belong to the genus *Wolbachia* with higher *p* values ranging from 0.0017 to 0.0452 (Additional file 11).

## Discussion

We performed viral metagenomics on pooled and individual *Ae*. *aegypti* and *Cx*. *quinquefasciatus* collected from Guadeloupe, a Caribbean island where mosquito-borne diseases are a major public health issue. No significant difference of total mapped reads (Fig. 1b) or the proportion of each taxonomic category (Fig. 1a, c) between single and pooled mosquitoes were observed, which proves the feasibility to use the NetoVIR protocol for single mosquito for viral metagenomics. To the best of our knowledge, all published studies on viral metagenomics of mosquito have been performed on pooled samples (see, e.g., [9–14]). With respect to novel virus exploration or arboviruses monitoring, it is indeed more effective to use pooled mosquitoes considering the tiny size and huge population of mosquitoes. However, the results from virome studies on pooled mosquitoes should be treated with caution, because the results could be strongly influenced by a single or limited number of mosquitoes acutely infected by a particular virus, or by the accidental pooling of mosquitoes from different (yet unknown) mosquito species, due to inaccurate morphology-based classification of mosquitoes. In this respect, a clinically relevant virus present in a low or medium viral load could be missed if pooled with one or more mosquitoes acutely infected with a clinically irrelevant virus. In our study, one unique sample categorized as *Ae*. *aegypti* by morphology-based classification was speculated to be a novel species through virome analysis (Figs. 3c, d and 4) and marker genes-based characterization (Fig. 7a). This mosquito possessed a eukaryotic virome distinct from *Ae*. *aegypti* and *Cx*. *quinquefasciatus* samples and contained multiple highly abundant very divergent novel viruses, and no known mosquito specific cox1 gene (except for some low cross reactivity with *Cx. bidens*) was detected.

A remarkable difference of the eukaryotic virome and phageome between *Ae*. *aegypti* and *Cx*. *quinquefasciatus* is revealed by our results. *Ae*. *aegypti* harbors a virome with higher abundance and diversity, mostly originated from the eukaryotic viruses. In contrast, more diverse bacteriophage contigs are abundantly present in *Cx*. *quinquefasciatus* compared to *Ae*. *aegypti* (Fig. 2). The qRT-PCR results consistently show lower eukaryotic viral concentration in *Cx*. *quinquefasciatus* (Fig. 6), supporting that the difference on eukaryotic viral abundance as identified by the NGS (Fig. 4) does not result from the bias of NGS sample preparation. The observation that both investigated mosquito species have distinct viromes, except for a few shared viral species (e.g., GMV and PCLPV) (Fig. 4), can be likely explained by the different habitat tropism, environmental factors (e.g., breeding sites and food resources), as well as selective pressures from the host like physicochemical conditions in gut [35], immune response [36], and microbiota interaction [37], which might also affect the viruses composition [38]. Although a “core virome” (loosely defined a set of viruses found in the majority of individuals in a particular mosquito population) seems present, the viral load can vary strongly between different individuals from the same species. However, it is striking that nearly identical viruses are found to infect a particular mosquito species across time (at least in two consecutive years) and space (different regions of Guadeloupe). Further surveillance will have to confirm the longer time stability of this mosquito-species specific core virome over longer periods of time and a larger geographic range.

Furthermore, the presence of the marker genes of *Wolbachia* sp. strain wPip (Fig. 7a) confirms previous observations about the broad distribution of *Cx*. *quinquefasciatus* populations with *Wolbachia* as endosymbiotic bacteria in Guadeloupe [39]. This study from Goindin and colleagues reported a 95.8% positive rate of *Wolbachia* sp. strain wPip-I infection in *Cx*. *quinquefasciatus* from Petit-Bourg, but none in *Ae*. *aegypti*. The *Wolbachia* endosymbionts of *Cx*. *quinquefasciatus* have shown to increase host resistance to West Nile virus (WNV) infection [40], possibly related to the production of small interfering RNAs [41]. Hence, we speculate that the lower copy number of eukaryotic viruses in *Cx*. *quinquefasciatus* might be a consequence of their colonization by *Wolbachia*. In contrast to the difference in virome between species, the qualitative virome within one species is surprisingly homogenous across different individuals of a species and across time, since nearly identical viruses were found in many individual mosquitoes, as well as in two consecutive collection years (Figs. 4, 5, and Additional file 7).

Although some of the discovered novel viruses (e.g., GMV, HTV, and GCTLV) were shown to be related to families/genera containing plant viruses (Fig. 5a, g), they cluster more closely with many unclassified viruses from a large study [24], which identified almost 1500 novel RNA viruses in invertebrates. This observation together with the identification of our novel viruses over different sampling sites and two consecutive years also strongly support that mosquitoes are their genuine host. Additionally, none of the novel viruses are closely related to known vector-borne pathogens of human or other mammals, suggesting that they are mosquito-specific. However, PCLPV which is highly prevalent in *Ae*. *aegypti* of Guadeloupe belongs to the genus *Phasivirus* (Fig. 5b), belonging to the same family (*Phenuiviridae*) containing the genus *Phlebovirus* harboring important human pathogens (e.g., Rift Valley fever virus). PCLPV also has been reported to be broadly disseminated in multiple organs (head, thorax, abdomen, legs, salivary gland, midgut, and ovary) of field-infected *Ae*. *aegypti* from China [42], and persistently infect *Ae*. *aegypti* cell lines [43]. Noticeably, a very divergent GMPV identified in the assumed new species sample Ab-AAF-1-3 is distantly related with known PCLPVs in the phylogeny (Fig. 5b), which indicated the possible adaption of this virus to its mosquito host. PCLPV does not infect vertebrate cells, due to the lack of NSs and NSm. NSs has been well established as the main phleboviral virulence determinant in the mammalian host [44] and NSm may play a role in the regulation of apoptosis [45]. However, a comprehensive characterization of novel lineages of insect-specific bunyaviruses with ancestral state reconstruction illustrated that the pathogenic bunyaviruses evolved from arthropod-specific progenitors [46]. Thus, viral metagenomics on mosquito can broaden our knowledge of viral composition and diversity in vectors, which will help us to explore the evolutionary history of insect-specific viruses and to predict the potential risk of spillover infection.

One major question arisen with the growing number of mosquito-specific viruses (MSVs) identified in recent years is how those viruses influence the transmission of pathogenic arboviruses to humans. The most well-studied MSV is the mosquito-specific flavivirus. However, the results of studies about the interaction between Culex flavivirus (CxFV) and WNV in live mosquitoes were inconclusive [16, 47], possibly because different mosquito species and viral strains were used. Furthermore, those studies did not investigate the potential persistent infection with other MSVs in the investigated mosquito strains, which could also have influenced their results. According to the observation of our qRT-PCR results, the viral load of four possible MSVs is variable among the individual mosquitoes within one species (Fig. 6a). The reason for this large observed variation is currently unknown, but might be very important to better understand vector competence. In light of the known arthropod antiviral mechanisms of superinfection exclusion [48, 49] or alteration of their immune system (e.g., RNA slicing and non-RNAi pathway [50–52]), we speculate that the viral load and therefore the vector competence for arboviral pathogens may vary significantly between individual mosquitoes from the same species, as we showed for different MSVs in our study. Due to the presence of multiple MSVs in a single mosquito (Fig. 6b), the influence of MSVs on vector competence might not result from a single virus independently but from the entire virome. Further studies need to be done to explore the effects of mosquito-specific virome on arbovirus transmission.

Since bacteria are known to be very important in the physiology of certain mosquitoes, we also characterized the phage population in the two mosquito species. Among the 194 confirmed phage contigs, 174 contigs were only present in *Cx*. *quinquefasciatus* samples, while *Ae*. *aegypti* only contained two unique contigs with 18 contigs shared by two mosquito species. Further correlation analyses of the 33 phage contigs longer than 1500 bp indicated that 24 of these contigs correlated with marker genes of *Wolbachia* sp. and *Cx*. *quinquefasciatus* (Fig. 7b). In the 24 contigs, 16 were confirmed to have *Wolbachia* as a most likely host based on k-mer-based predictions using WIsH (Additional file 11). It should be noted that it is very likely that the different identified contigs all belong to one or a limited number of phage genomes, most likely infecting *Wolbachia* species. Previous studies have shown that the effects of reproductive disorders in mosquitoes caused by *Wolbachia* partially depend on their phage infection status [53, 54]. *Wolbachia*-associated bacteriophages are believed to be the mobile genetic elements resulting in a high genetic diversity of *Wolbachia* [55–57], and proposed as a potential transformation tool for genetic modification of mosquito vectors [58]. The low abundance of phage contigs in *Ae*. *aegypti* is probably a reflection of the absence of endosymbiotic bacteria, or alternatively, (but less likely) is that they were too divergent to be detected using the approach followed in this study. The deeper understanding of tripartite (mosquito-bacteria-phage) interactions will help the development of novel biological vector control. In addition, the correlation analysis and WIsH prediction performed in our study are providing us a glimpse of the relationship between phage sequences and prokaryotic host. Since our study was designed for virome analysis, only the (small) bacteria whose genomic DNA survived the NetoVIR protocol could be identified through marker gene detection. Due to the lack of the bacterial genomes from our samples, the WIsH prediction can only run on the selected bacterial genomes from database, which will fail to predict the host of novel phage sequences if the host bacterium is not present in the bacterial dataset. The three phage contigs of *Cystoviridae* (Fig. 7b) whose natural host is the genus *Pseudomonas* (https://viralzone.expasy.org/165) had highest log-likelihood with *Pseudomonas savastanoi* among the tested 37 bacterial genomes, but the *p* values were only around 0.4 (Additional file 11), which suggests that the genome of the host *Pseudomonas* strain (or another bacterium) present in our mosquito samples is rather divergent compared to those in the database. The bacterial composition and genomes in the mosquitoes need to be further explored by bacteria-specific 16S rRNA sequencing and metagenome shotgun sequencing, which will help to confirm the predicted relationship between phage sequences and bacteria.

## Conclusions

Our study firstly demonstrates that viral metagenomics is feasible on single mosquitoes. Interspecific comparisons show striking differences in abundance and diversity between the viromes of *Ae*. *aegypti* and *Cx*. *quinquefasciatus*. Many viruses are found to be present in multiple mosquitoes of the same species over different sampling sites and two consecutive years, suggesting that each species might have their own rather stable “core eukaryotic virome”. This needs to be further confirmed with larger-scale sampling from additional sites and time points. Additionally, we discover 11 novel eukaryotic viruses, which are speculated to be mosquito-specific. *Wolbachia sp.* strain wPip was found to be prevalent in *Culex quinquefasciatus* and a number of associated phage sequences are identified. This study reveals precise virome composition data (including eukaryotic viruses and bacteriophages) of the two most common mosquito species in Guadeloupe through viral metagenomic analysis on individual mosquitoes. How the interaction between viruses and host interferes the physiology and vector competence of mosquitoes needs to be further studied.

## Methods

### Mosquito collection and pooling information

*Ae. aegypti* and *Cx. quinquefasciatus* were collected as adults in August–September 2016 (wet season) and May–June 2017 (end of dry season) in households from the east and west island of Guadeloupe (Additional file 1). After collection, mosquito species were determined by morphological identification under a binocular loupe at a magnification of × 56 (Leica M80, Leica, Nanterre, France) using morphological descriptions [59, 60] and stored at − 80 °C until use. A total number of 95 mosquito sampled in Les Abymes of 2016 were grouped into four pools for sequencing: male and female of *Ae*. *aegypti* and *Cx*. *quinquefasciatus* (Table 1). For the samples collected in 2017, we sequenced six pools for each species, gender, and sampling location: five pools with single mosquito and one pool containing five mosquitoes, with 36 pools in total (Table 1). Furthermore, a negative control (PBS), which was processed together with other mosquito pools following the same procedure, was also sequenced.

### Sample processing and sequencing

An optimized sample preparation protocol for viral metagenomics—NetoVIR [61] was used to analyze the mosquito pools and individuals as well as a negative control. Briefly, whole mosquitoes were homogenized with 200 μl PBS in a MINILYS tissue homogenizer for 1 min at 3000 rpm using 2.8 nm ceramics beads (Precellys) and centrifuged (17,000 g for 3 min), and 150 μl supernatant were then used for filtration (0.8 μm pore size) to enrich for viral particles. The filtrate was then treated with a cocktail of Benzonase (Novagen) and Micrococcal Nuclease (New England Biolabs) in a homemade buffer (1 M Tris, 100 mM CaCl_2_, and 30 mM MgCl_2_) to digest free-floating nucleic acids. DNA and RNA were extracted (QIAGEN Viral RNA mini kit), reverse-transcribed, and randomly amplified using a slightly modified Whole Transcriptome Amplification 2 (WTA2) Kit procedure (Sigma-Aldrich). WTA2 products were purified, and the libraries were prepared for Illumina sequencing using the NexteraXT Library Preparation Kit (Illumina). A cleanup after library synthesis was performed using a 1.8 ratio of Agencourt AMPure XP beads (Beckman Coulter, Inc.). Sequencing of the samples was performed on a NextSeq500 High throughput platform (Illumina) for 300 cycles (2 × 150 bp paired ends) (Additional file 2).

### Bioinformatic analysis of eukaryotic virome and phageome

The obtained raw paired-end reads were trimmed for quality and adapters using Trimmomatic [62]. Reads mapping to a set of contaminating contigs known to be present in the negative controls (contamination of reagents) were removed using BWA [63] and the remaining reads are de novo assembled into contigs using SPAdes [64]. Contigs from all pools longer than 500 bp were clustered to remove redundancy at 95% nucleotide identity over 80% of the length using ClusterGenomes (https://bitbucket.org/MAVERICLab/docker-clustergenomes). These non-redundant (nr) contigs collection was classified using DIAMOND [65] against the nr database on sensitive mode for taxonomic annotation. KronaTools [66] were used to parse the output file of DIAMOND, which found the least common ancestor of the best 25 DIAMOND hits (based on BLASTx score) for each contig. All contigs annotated as eukaryotic virus were extracted using an in-house python script. Bacteriophages were identified using combined approaches including BLASTn [67], DIAMOND, as well as MetaPhinder2 (ANI ≥ 10%) [68] and VirSorter (category 1 and 2) [69]. Hmmsearch against the Prokaryotic Virus Orthologous Groups (pVOGs), eggNOG-mapper, and PfamScan were further used to confirm the bacteriophage contigs identified by MetaPhinder2 and VirSorter. The contigs without pVOGs hits or phage-associated proteins/domains were classified to bacteriophage to be confirmed (bacteriophageTBC). Individual pool magnitudes were obtained by mapping trimmed and decontaminated reads to the nr contigs collection using BBMap (https://github.com/BioInfoTools/BBMap). Abundance tables for eukaryotic viruses and bacteriophages were extracted respectively and further used for ecological analysis in R with the ggplot2 [70], phyloseq [71], metagenomeSeq [72], microbiomeSeq (https://github.com/umerijaz/microbiomeSeq), and vegan [73] packages.

### Virus identification and phylogenetic analysis

ORF Finder was used to identify ORFs in the obtained eukaryotic viral contigs, and contigs believed to represent the complete coding capacity of a viral genome were selected [74]. To identify different variants of these viruses in the individual samples, the trimmed and decontaminated reads of individual samples and pools were mapped to those selected genomes (Table 2). Amino acid sequences of RdRp or PB1 were used to determine the evolutionary history of the discovered viruses together with appropriate reference strains from GenBank. Alignments of the viral RdRp or PB1 were performed with MAFFT v7.222 [75] using the E-INS-I algorithm. Ambiguously aligned regions were then removed by trimAl v1.2 [76] using automated trimming heuristic, which is optimized for maximum likelihood (ML) phylogenetic tree reconstruction. The best-fit model of amino acid substitution was subsequently determined by ProtTest v3.4.2 [77]. ML phylogenetic trees are constructed by PhyML v3.1 [78], employing the best-fit substitution model and Subtree Pruning and Regrafting (SPR) as tree topology improvement algorithm. Branch supports were computed by approximate likelihood ratio test (aLRT) with SH-like support as implemented in PhyML. Phylogenetic trees were drawn from the PhyML results with FigTree v1.4.3 (https://github.com/rambaut/figtree/releases).

### Prevalence of core viruses

Seventy-two *Ae*. *aegypti* and 24 *Cx*. *quinquefasciatus* individuals collected in 2017 (Table 3) were used to determine the prevalence of a selection of highly abundant viruses. These viruses were Phasi charoen-like phasivirus (PCLPV), Guadeloupe mosquito virus (GMV), Guadeloupe Aedes aegypti totivirus (GAATV), Aedes aegypti anphevirus (AANV), Guadeloupe Culex rhabdovirus (GCRV), and Guadeloupe Culex tymo-like virus (GCTLV). The specific primers and probes of each virus (Additional file 10)were designed from the alignment of all nearly complete genomes recovered from NGS data using GenScript Real-time PCR (TaqMan) Primer Design (https://www.genscript.com/tools/real-time-pcr-tagman-primer-design-tool). Viral RNA was isolated from individual mosquitoes, eluted in 60 μl elution buffer (QIAGEN Viral RNA mini kit) and subsequently tested for each virus by qRT-PCR in duplicate. The qRT-PCRs were run in 20 μl reaction volumes with 5 μl TaqMan Fast Virus 1-Step Master Mix (ThermoFisher), 2 μl forward and reverse primer (10 μM), 1 μl probe (5 μM), and 5 μl viral RNA extraction of samples. The standards (oligonucleotides ordered from Eurogentec) with known concentration were used to establish a calibration curve through serial tenfold dilutions (10^9^ to 10^2^ copies), subsequently used for calculation of viral concentration. The total copies of each virus per mosquito were determined by multiplying the qRT-PCR result by 12 (dilution factor: 5 μl out of 60 μl viral RNA extraction of mosquito was used for qRT-PCR).

### Marker genes detection and correlation analysis

All cytochrome c oxidase I (cox1), DNA gyrase subunit B (gyrB), and recombinase A protein (recA) genes were downloaded from NCBI, and then the redundant and cox1 genes of mammals were removed. The trimmed and decontaminated reads of individual pools were mapped against each gene database using BBMap. The mapped read numbers were normalized for the reads per kilobase million (RPKM). Briefly, total number of reads in a sample are divided by 1,000,000 resulting in a “per million” scaling factor. The mapped read counts are divided by the “per million” scaling factor to get the RPM and then RPM values are divided by the length of the gene to give the RPKM value. The marker genes whose sum RPKM value across all samples was higher than 0.001 were shown in the heatmap (Fig. 7a) and further used for the correlation analysis (Fig. 7b).

The relative abundance of the 33 phage contigs (longer than 1500 bp), bacterial and mosquito marker genes in each sample were calculated by dividing the reads number mapped to contigs or genes to total reads number of each sample. The bacterial marker genes included the recA, gyrB, and cox1 genes of *Chromobacterium violaceum*, *Cupriavidus taiwanensis*, *Wolbachia endosymbiont of Culex quinquefasciatus Pel wPip strain*, *Wolbachia endosymbiont of Drosophila melanogaster*, and *Wolbachia endosymbiont of Drosophila simulans wNo*. Then the abundance table was used for correlation analysis with the corrplot package [79]. A matrix of Pearson’s r rank correlation coefficients was computed for all possible pairs of bacteriophage contigs and marker genes. Ranks were computed using efficient algorithms, using midranks for ties. *P* values were approximated by using the *t* or *F* distributions and corrected for multiple comparisons with Holm's method.

For host prediction with WIsH [34], 37 bacterial genomes were downloaded from NCBI, which included all strains in the genus *Wolbachia* and *Chromobacterium*, eight strains in genus *Cupriavidus*, seven strains in genus *Pseudomonas*, and five randomly selected strains (Additional file 11). A Markov model was created from each bacterial genome. In order to calculate the *p* value, the parameters of Gaussian null distribution for each model need to be given as input. For the bacterial strains whose null parameters were not provided by WIsH, a set of 1420 phage genomes known not to infect the strains [34] were used to run the predictions for each bacterial genome and prediction likelihood was used to fit the null-model parameters. The parameters for the associated null-models were computed with WIsH provided script (computeNullParameters.R). Then we run the prediction on 33 phage contigs (> 1500 bp) identified in this study and 30 phage contigs from RefSeq Virus database and used *p* value < 0.001 as threshold (Additional file 11) [34].

## Additional files

Additional file 1: Sampling sites in Guadeloupe. (PDF 4193 kb)

Additional file 2: Sequencing information and SRA number. (XLSX 13 kb)

Additional file 3: Comparison between reads proportion for each taxomomic category in *Aedes aegypti* and *Culex quinquefasciatus* per sample/pool without sample Ab-AAF-1-3. (PDF 403 kb)

Additional file 4: Alpha diversity of eukaryotic viruses on species level between gender or locations. (PDF 428 kb)

Additional file 5: Alpha and beta diversity of the virome in *Aedes aegypti* and *Culex quinquefasciatus* samples/pools without sample Ab-AAF-1-3. (PDF 455 kb)

Additional file 6: The longest viral genome length of each species identified in this study and their related the reference genomes length as well as the accession number. (XLSX 16 kb)

Additional file 7: Relative abundance of virus species identified from 2016 samples. (PDF 1480 kb)

Additional file 8: Genome organization of novel viruses. (PDF 1470 kb)

Additional file 9: Phylogenetic tree of RdRp and capsid protein of GAATVs identified in this study. (PDF 433 kb)

Additional file 10: qRT-PCR primers, probes and conditions. (XLSX 11 kb)

Additional file 11: Null parameters of bacterial models and WIsH prediction results. (XLSX 17 kb)

## Data Availability

The raw sequencing datasets for the current study are available in the NCBI Sequence Read Archive (SRA) repository, under the Bioproject with accession code PRJNA515586 (www.ncbi.nlm.nih.gov/bioproject/515586). Sequence files, metadata, and R script used for analysis in this study have been deposited in Figshare (https://figshare.com/projects/Guadeloupe_mosquito_virome/67049).

## Abbreviations

AANV: Aedes aegypti anphevirus
AAOLV: Aedes alboannulatus orthomyxi-like virus
*Ae*. *aegypti*: *Aedes aegypti*
bacteriophageTBC: Bacteriophage to be confirmed
BmMLV: *Bombyx mori* Macula-like virus
CMLV2: Culex mononega-like virus 2
cox1: cytochrome c oxidase subunit 1
*Cx. quinquefasciatus*: *Culex quinquefasciatus*
GAATLV: Guadeloupe Aedes aegypti toti-like virus
GAATV: Guadeloupe Aedes aegypti totivirus
GCRV: Guadeloupe Culex rhabdovirus
GCTLV: Guadeloupe Culex tymo-like virus
GMMLV 1/2: Guadeloupe mosquito mononega-like viruses 1/2
GMPV: Guadeloupe mosquito phasivirus
GMQLV1/2/3: Guadeloupe mosquito quaranja-like virus 1/2/3
GMV: Guadeloupe mosquito virus
gyrB: DNA gyrase subunit B
HMV2: Hubei mosquito virus 2
HTV: Humaita-Tubiacanga virus
ISVs: Insect-specific viruses
MSVs: Mosquito-specific viruses
NMDS: Non-metric multi-dimensional scaling
ORFs: Open reading frames
PCLPV: Phasi Charoen-like phasivirus
PERMANOVA: Permutational multivariate analysis of variance
pVOGs: Prokaryotic Virus Orthologous Groups
qRT-PCR: quantitative real-time RT-PCR
RdRp: RNA-dependent RNA polymerase
recA: recombinase A protein
RPKM: Reads per kilobase million
WMV9: Wuhan mosquito virus 9
WNV: West Nile virus
WMV4: Wuhan Mosquito Virus 4
WMV6: Wuhan Mosquito Virus 6
WSLV4: Wenzhou sobemo-like virus 4
