# Supplementary figures and images for: Stable distinct core eukaryotic viromes in different mosquito species from Guadeloupe, using single mosquito viral metagenomics
Source: Microbiome. 2019 Aug 28;7:121. doi: 10.1186/s40168-019-0734-2 (PMC6714450; doi:10.1186/s40168-019-0734-2)

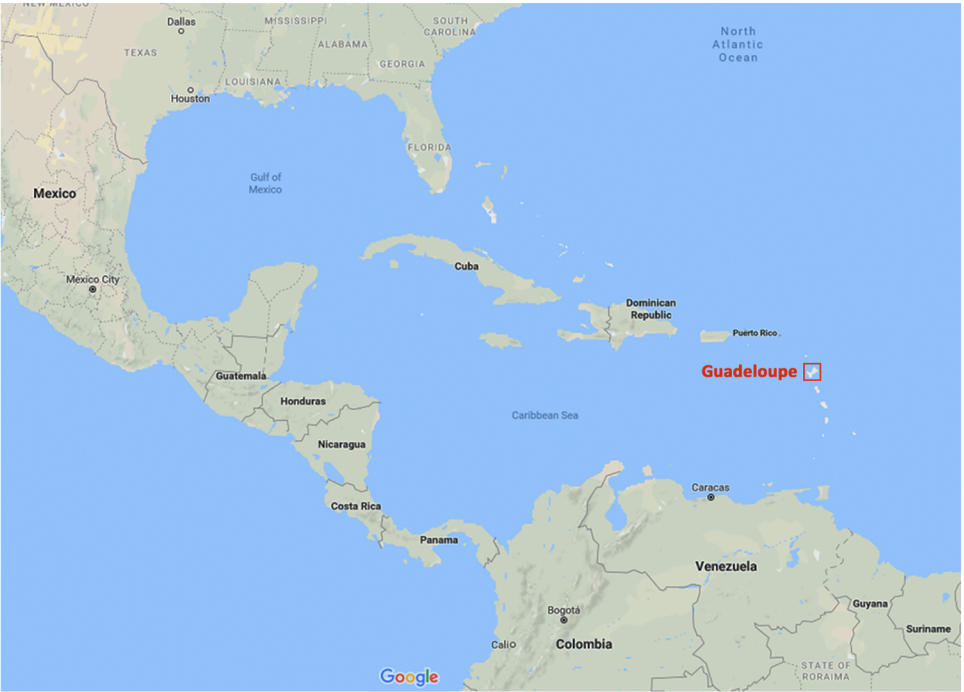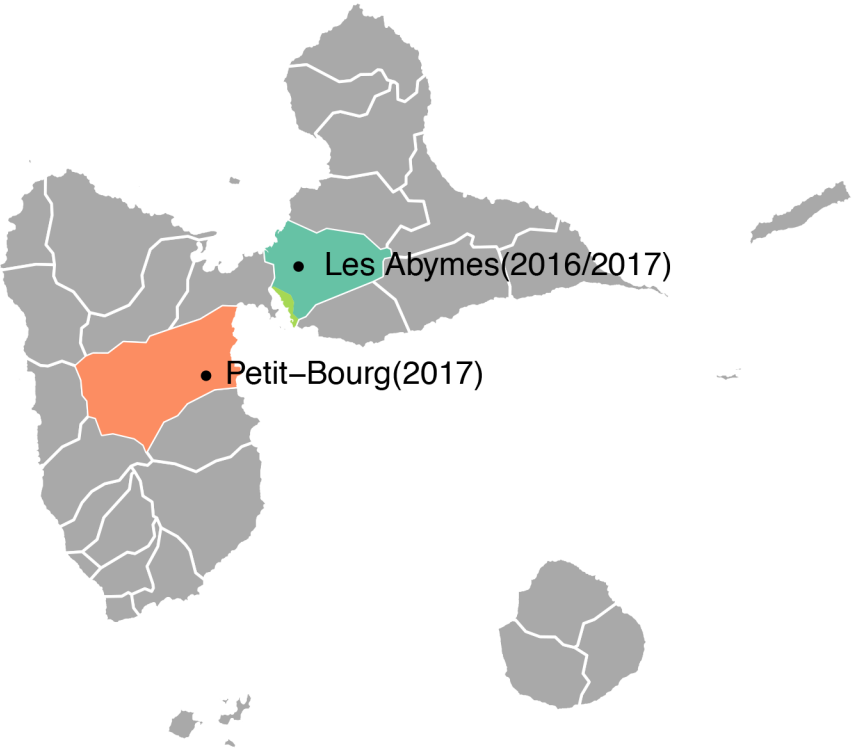

Additional file 1. Sampling sites of Guadeloupe in 2016 and 2017

Supplement: Supplementary file 1 — Sampling sites in Guadeloupe. (PDF 4193 kb) [file 40168_2019_734_MOESM1_ESM.pdf]
