# Supplementary material for: Stable distinct core eukaryotic viromes in different mosquito species from Guadeloupe, using single mosquito viral metagenomics
Source: Microbiome. 2019 Aug 28;7:121. doi: 10.1186/s40168-019-0734-2 (PMC6714450; doi:10.1186/s40168-019-0734-2)
Supplement: Supplementary file 3 — Comparison between reads proportion for each taxomomic category in Aedes aegypti and Culex quinquefasciatus per sample/pool without sample Ab-AAF-1-3. (PDF 403 kb) [file 40168_2019_734_MOESM3_ESM.pdf]

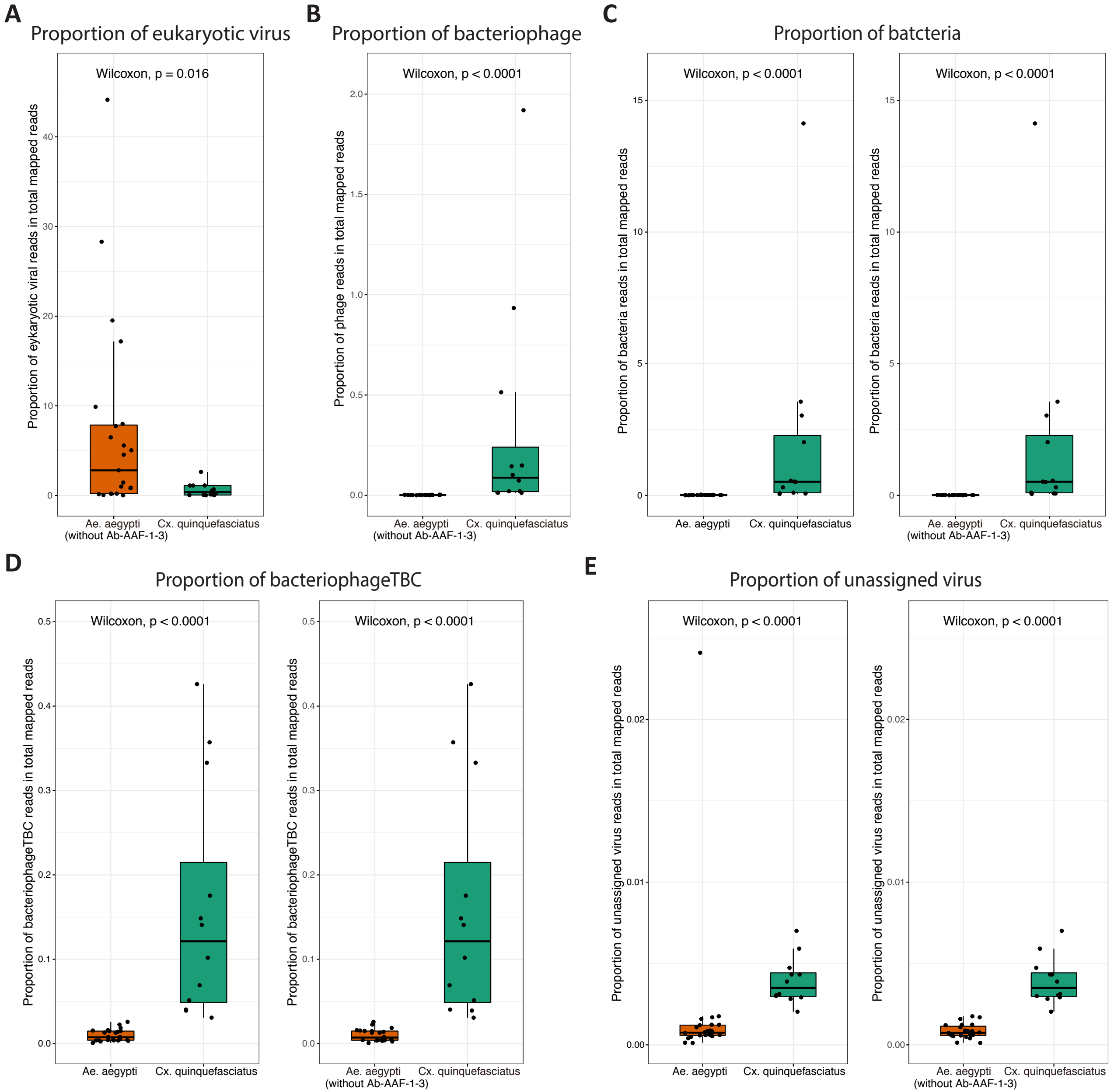


Additional file 3: Comparison between reads proportion for each taxomomic category in *Aedes aegypti* and *Culex quinquefasciatus* per sample/pool without sample Ab-AAF-1-3. (A) Comparison of the proportion of eukaryotic virus reads in the two mosquito species without sample Ab-AAF-1-3. (B) Comparison of the proportion of bacteriophage reads in the two mosquito species without sample Ab-AAF-1-3. (C) Comparison of the proportion of bacteria reads in the two mosquito species with and without sample Ab-AAF-1-3. (D) Comparison of the proportion of bacteriophageTBC reads in the two mosquito species with and without sample Ab-AAF-1-3. (E) Comparison of the proportion of unassigned virus reads in the two mosquito species with and without sample Ab-AAF-1-3.
