# Supplementary material for: Stable distinct core eukaryotic viromes in different mosquito species from Guadeloupe, using single mosquito viral metagenomics
Source: Microbiome. 2019 Aug 28;7:121. doi: 10.1186/s40168-019-0734-2 (PMC6714450; doi:10.1186/s40168-019-0734-2)
Supplement: Supplementary file 4 — Alpha diversity of eukaryotic viruses on species level between gender or locations. (PDF 428 kb) [file 40168_2019_734_MOESM4_ESM.pdf]

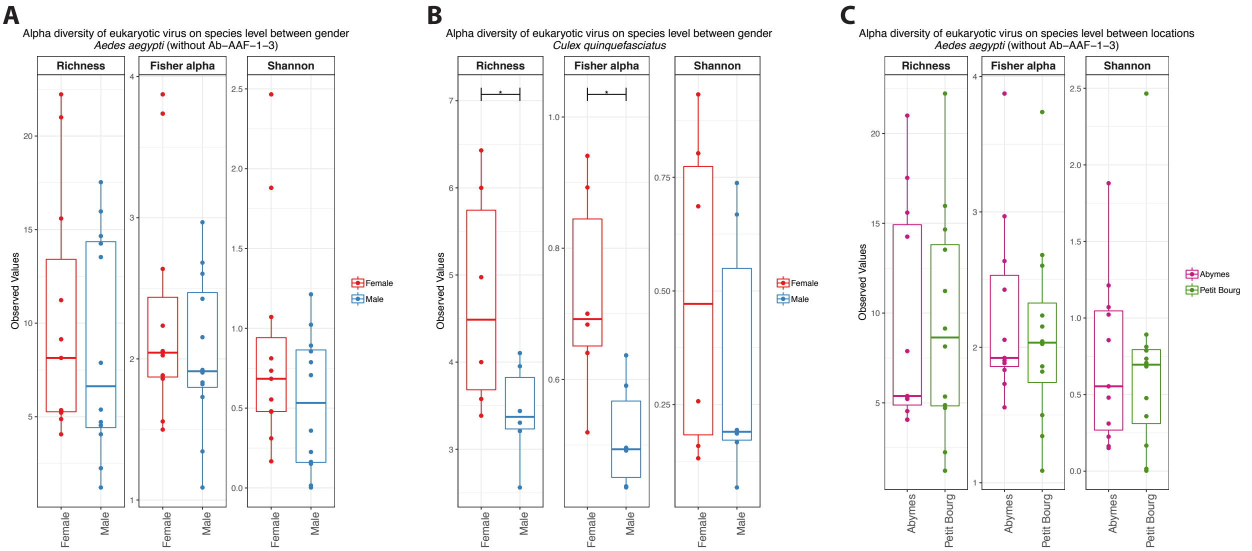

Additional file 4: Alpha diversity of eukaryotic viruses on species level between gender or locations. (A) Alpha diversity comparison on eukaryotic viral species between gender of *Aedes aegypti*. (B) Alpha diversity comparison on eukaryotic viral species between gender of *Culex quinquefasciatus*. (C) Alpha diversity comparison on eukaryotic viral species between two collected locations of *Aedes aegypti*. Pairwise ANOVA: $p < 0.01$ (*), $p < 0.001$ (**), $p < 0.0001$ (***)
