# Supplementary material for: Stable distinct core eukaryotic viromes in different mosquito species from Guadeloupe, using single mosquito viral metagenomics
Source: Microbiome. 2019 Aug 28;7:121. doi: 10.1186/s40168-019-0734-2 (PMC6714450; doi:10.1186/s40168-019-0734-2)
Supplement: Supplementary file 5 — Alpha and beta diversity of the virome in Aedes aegypti and Culex quinquefasciatus samples/pools without sample Ab-AAF-1-3. (PDF 455 kb) [file 40168_2019_734_MOESM5_ESM.pdf]

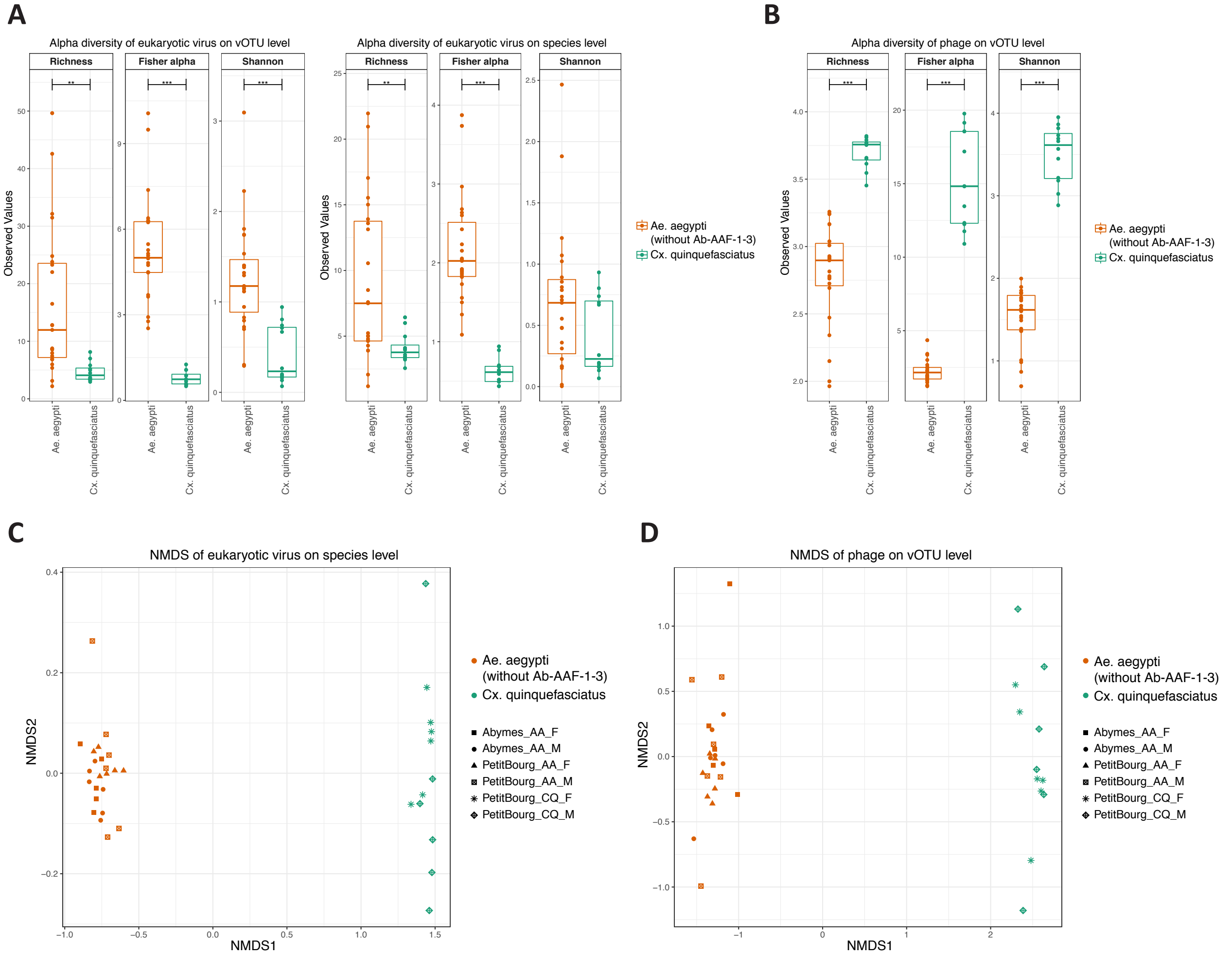

Additional file 5. Alpha and beta diversity of the virome in *Aedes aegypti* and *Culex quinquefasciatus* samples/pools without sample Ab-AAF-1-3. (A) Alpha diversity of eukaryotic viruses in *Ae. aegypti* and *Cx. quinquefasciatus* on vOTU and species level without sample Ab-AAF-1-3. (B) Alpha diversity of bacteriophages in *Ae. aegypti* and *Cx. quinquefasciatus* on vOUT level without sample Ab-AAF-1-3. Pairwise ANOVA: p < 0.01 (*), p < 0.001 (**), p < 0.0001 (***). (C) Non-metric multidimensional scaling (NMDS) of eukaryotic viruses on viral species level without sample Ab-AAF-1-3. PERMANOVA test on mosquito species: p = 0.001, R^2 = 0.129. (D) NMDS of bacteriophages on vOTU level without sample Ab-AAF-1-3. PERMANOVA test on mosquito species: p = 0.001, R^2 = 0.324.
