# Supplementary material for: Stable distinct core eukaryotic viromes in different mosquito species from Guadeloupe, using single mosquito viral metagenomics
Source: Microbiome. 2019 Aug 28;7:121. doi: 10.1186/s40168-019-0734-2 (PMC6714450; doi:10.1186/s40168-019-0734-2)
Supplement: Supplementary file 7 — Relative abundance of virus species identified from 2016 samples. (PDF 1480 kb) [file 40168_2019_734_MOESM7_ESM.pdf]

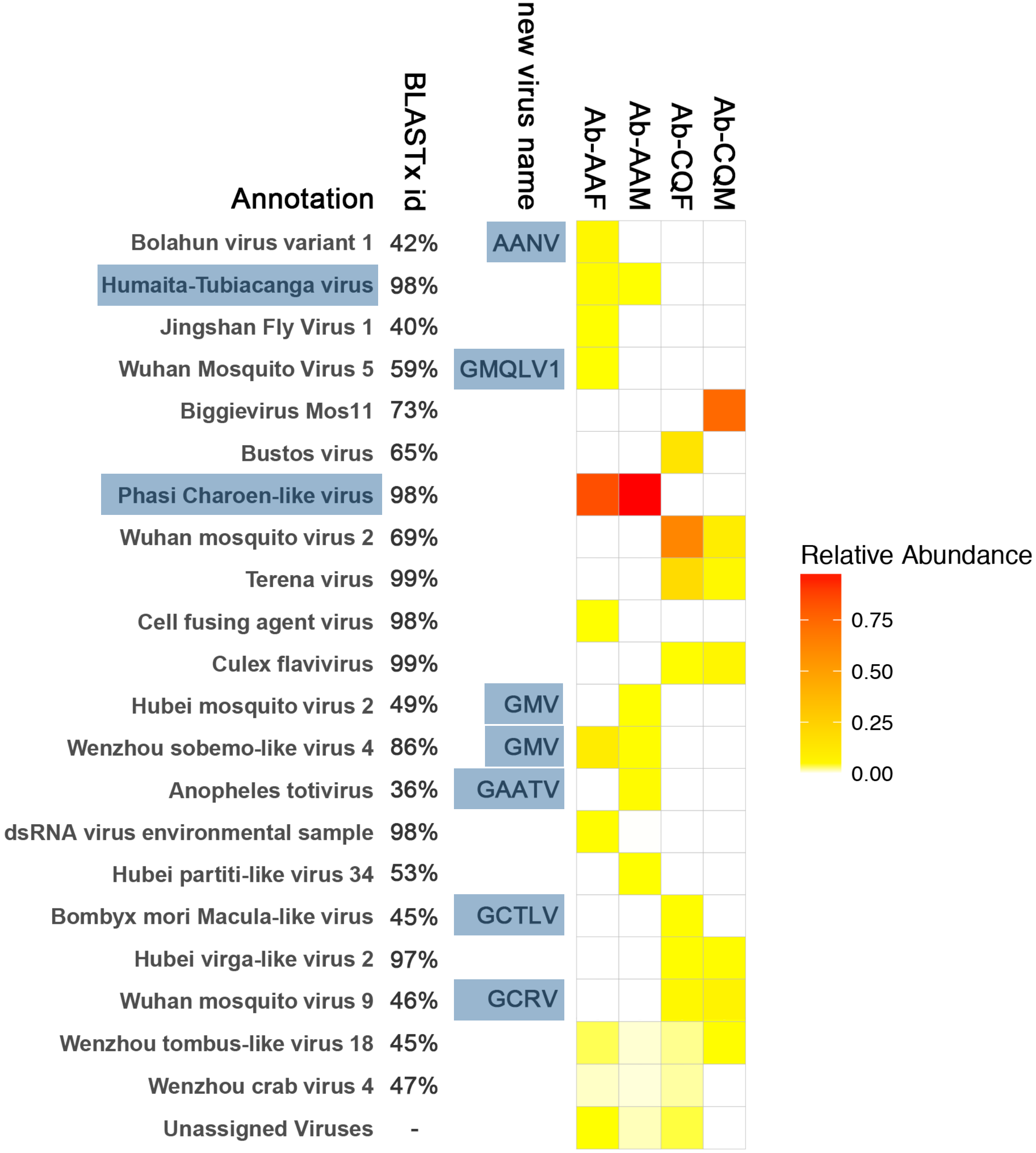

Additional file 7: Relative Abundance of Virus Species identified from 2016 samples

The viral species names shown in the heatmap are from the result of annotation tools DIAMOND and KronaTools. Viruses also identified in 2017 were marked with blue shade.For each of the contigs assigned to a particular species, the ORF with the highest BLASTx identity to a reference sequence was taken, and the average identity of these different ORFs is shown in the shaded blue boxes.
