# Supplementary material for: Stable distinct core eukaryotic viromes in different mosquito species from Guadeloupe, using single mosquito viral metagenomics
Source: Microbiome. 2019 Aug 28;7:121. doi: 10.1186/s40168-019-0734-2 (PMC6714450; doi:10.1186/s40168-019-0734-2)
Supplement: Supplementary file 8 — Genome organization of novel viruses. (PDF 1470 kb) [file 40168_2019_734_MOESM8_ESM.pdf]

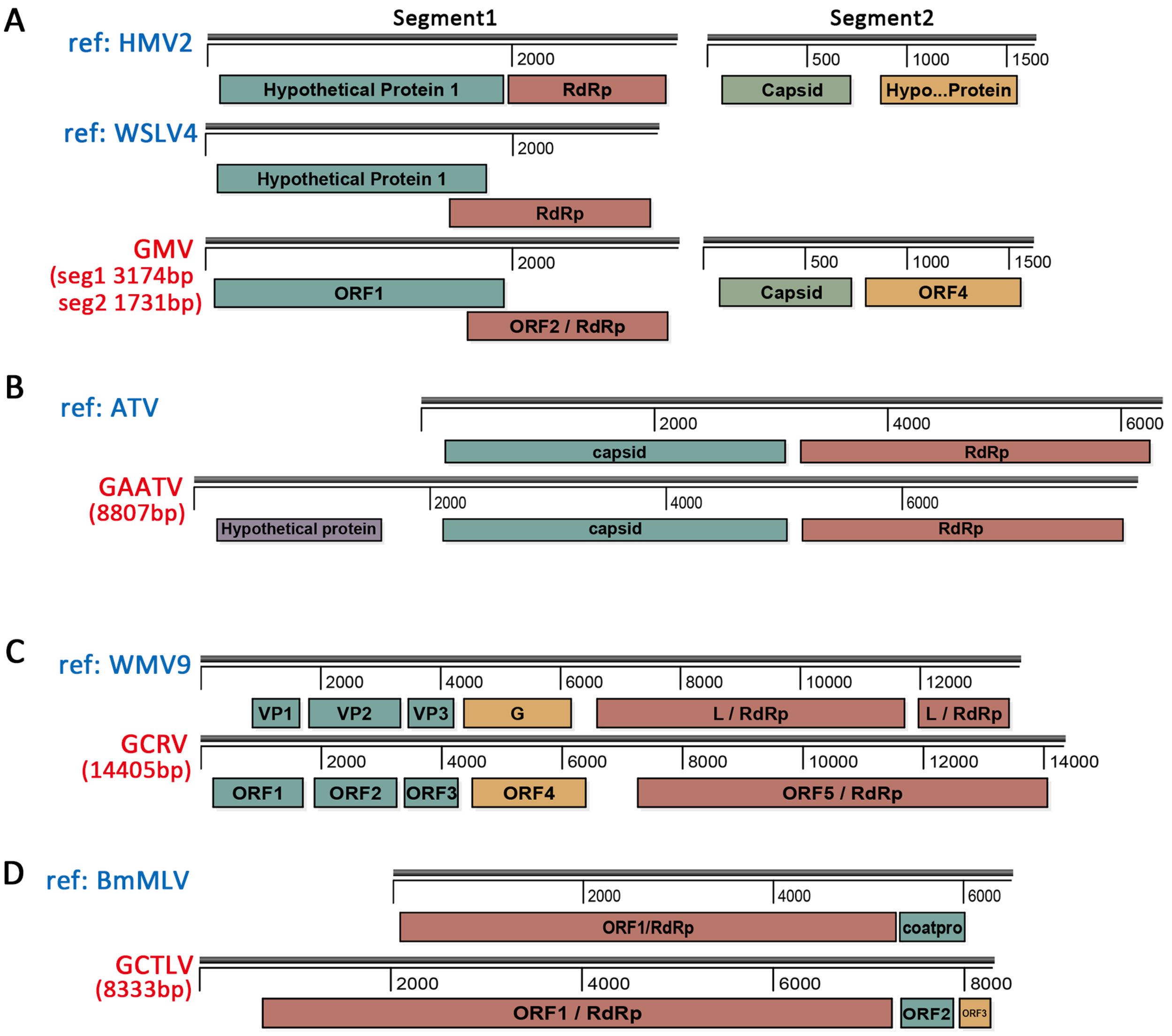

Additional file 8: Genome organization of novel viruses and their cloest references.

HMV2 - Hubei mosquito virus 2
GMV - Guadeloupe mosquito virus
GAATV - Guadeloupe Aedes aegypti totivirus
GCRV - Guadeloupe Culex rhabdovirus
GCTLV - Guadeloupe Culex tymo-like virus
WSLV4 - Wuhan sobemo-like virus 4
ATV - Anopheles totivirus
WMV9 - Wuhan mosquito virus 9
BmMLV - Bombyx mori Macula–like virus
