# Supplementary material for: Stable distinct core eukaryotic viromes in different mosquito species from Guadeloupe, using single mosquito viral metagenomics
Source: Microbiome. 2019 Aug 28;7:121. doi: 10.1186/s40168-019-0734-2 (PMC6714450; doi:10.1186/s40168-019-0734-2)
Supplement: Supplementary file 9 — Phylogenetic tree of RdRp and capsid protein of GAATVs identified in this study. (PDF 433 kb) [file 40168_2019_734_MOESM9_ESM.pdf]

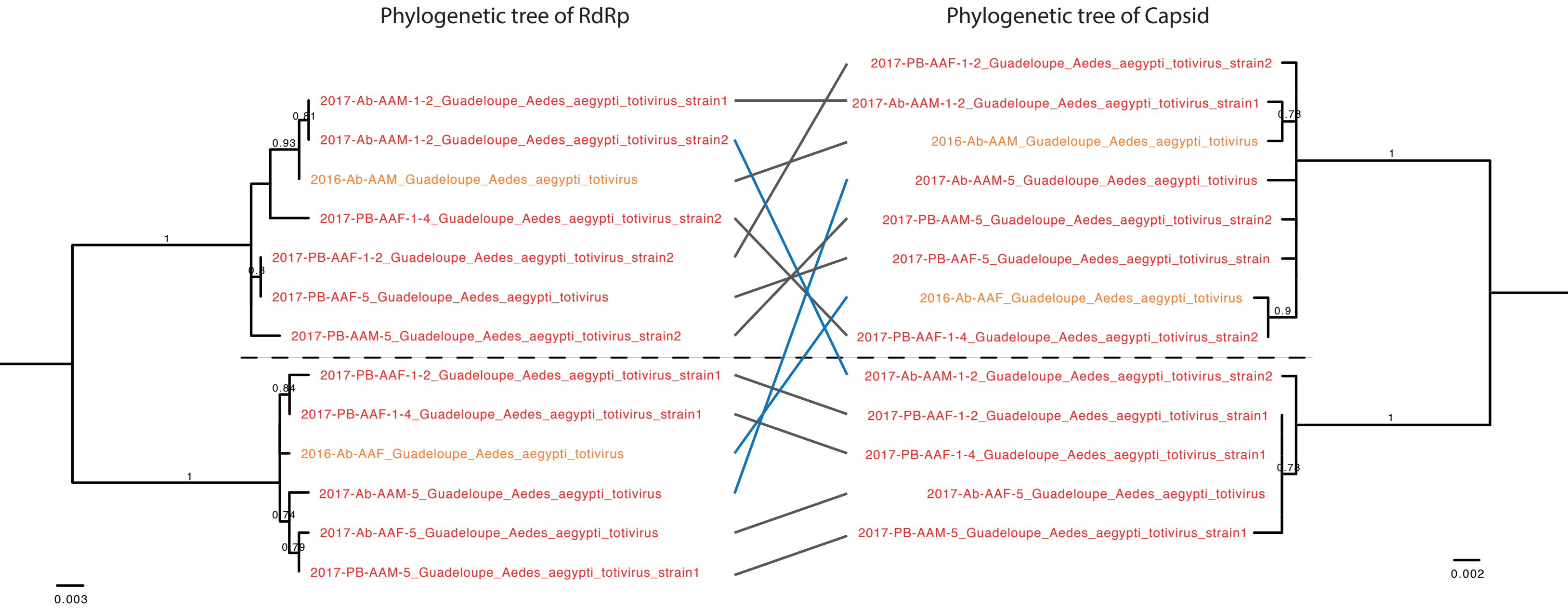

Additional file 9: Phylogenetic tree of RdRp and capsid protein of GAATVs identified in this study. The GAATVs linked by grey or blue lines fall into the same or different cluster(s).
